# RNAi-mediated gene knockdown of *progesterone 5β-reductases* in *Digitalis lanata* reduces 5β-cardenolide content

**DOI:** 10.1007/s00299-021-02707-3

**Published:** 2021-06-19

**Authors:** Jan Klein, Elisa Horn, Mona Ernst, Tim Leykauf, Tamara Leupold, Maja Dorfner, Laura Wolf, Anastasiia Ignatova, Wolfgang Kreis, Jennifer Munkert

**Affiliations:** Department of Biology, University of Erlangen-Nuremberg, 91058 Erlangen, Germany

**Keywords:** Progesterone 5β-reductases (P5βR), Cardenolide biosynthesis, *Digitalis lanata*, RNAi-mediated knockdown, Glutathione, Reactive electrophile species

## Abstract

**Key message:**

**Studying RNAi-mediated** ***DlP5βR1*** **and** ***DlP5βR2*** **knockdown shoot culture lines of** ***Digitalis lanata,*** **we here provide direct evidence for the participation of PRISEs (progesterone 5β-reductase/iridoid synthase-like enzymes) in 5β-cardenolide formation.**

**Abstract:**

Progesterone 5β-reductases (P5βR) are assumed to catalyze the reduction of progesterone to 5β-pregnane-3,20-dione, which is a crucial step in the biosynthesis of the 5β-cardenolides. P5βRs are encoded by *VEP*1-like genes occurring ubiquitously in embryophytes. P5βRs are substrate-promiscuous enone-1,4-reductases recently termed PRISEs (progesterone 5β-reductase/iridoid synthase-like enzymes). Two PRISE genes, termed *DlP5βR1* (AY585867.1) and *DlP5βR2* (HM210089.1) were isolated from *Digitalis lanata*. To give experimental evidence for the participation of PRISEs in 5β-cardenolide formation, we here established several RNAi-mediated *DlP5βR1* and *DlP5βR2* knockdown shoot culture lines of *D. lanata*. Cardenolide contents were lower in *D. lanata P5βR-RNAi* lines than in wild-type shoots. We considered that the gene knockdowns may have had pleiotropic effects such as an increase in glutathione (GSH) which is known to inhibit cardenolide formation. GSH levels and expression of glutathione reductase (GR) were measured. Both were higher in the *Dl P5βR-RNAi* lines than in the wild-type shoots. Cardenolide biosynthesis was restored by buthionine sulfoximine (BSO) treatment in *Dl P5βR2-RNAi* lines but not in *Dl P5βR1-RNAi* lines. Since progesterone is a precursor of cardenolides but can also act as a reactive electrophile species (RES), we here discriminated between these by comparing the effects of progesterone and methyl vinyl ketone, a small RES but not a precursor of cardenolides. To the best of our knowledge, we here demonstrated for the first time that *P5βR1* is involved in cardenolide formation. We also provide further evidence that PRISEs are also important for plants dealing with stress by detoxifying reactive electrophile species (RES).

**Supplementary Information:**

The online version contains supplementary material available at 10.1007/s00299-021-02707-3.

## Introduction

The successful introduction of foxglove extracts to treat congestive heart failure in the eighteenth century (Withering 1941) made plants from the genus *Digitalis* (Plantaginaceae) worth investigating (Luckner and Wichtl 2000). In the twentieth century cardiac glycosides, the bioactive principles of *Digitalis* plants replaced the use of extracts. Although other medications have superseded *Digitalis* in the treatment of heart failure, cardenolides came back into focus as they showed anti-viral and anti-tumoral activity (Prassas and Diamandis 2008; Bertol et al. 2011; Schneider et al. 2017). Today cardenolides such as digoxin, digitoxin, and lanatoside C are still isolated from *Digitalis lanata* Ehrh. leaves (Luckner and Wichtl 2000). This time-consuming process is necessary as the chemical synthesis of cardenolides is not economically feasible. Only parts of their biosynthesis *in planta* are known which makes it difficult to design alternative biotechnological production systems using yeast or bacteria (Rieck et al. 2019).

**Fig. 1:**
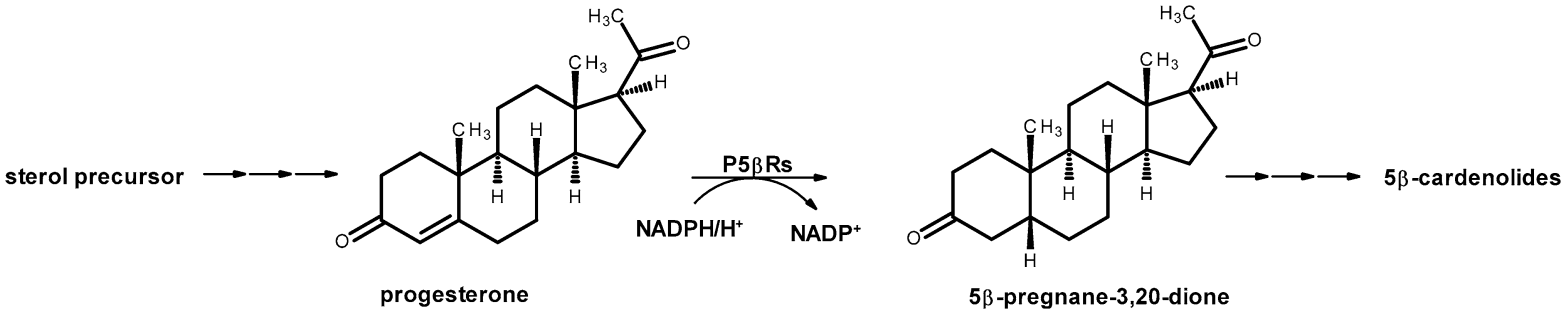
Putative biosynthetic pathway of 5β-cardenolides in *Digitalis*. Progesterone 5β-reductases catalyze the stereospecific reduction of progesterone to 5β-pregnane-3,20-dione under the consumption of NADPH/H^+^

A crucial step in the biosynthesis of 5β-cardenolides is the reduction of progesterone to 5β-pregnane-3,20-dione which is assumed to be catalyzed by progesterone 5β-reductases (Gärtner et al. 1994; Herl et al. 2006; Fig. 1). However, direct proof was not provided so far. Enzymes with P5βR activity occur in many cardenolide-containing and cardenolide-free plants (Bauer et al. 2010). For example, a steroid 5β-reductase (St5βR) with a progesterone 5β-reductase activity much higher than that of *D. lanata* was identified in the cardenolide-free plant *Arabidopsis thaliana* (Herl et al. 2009). Moreover, P5βR-like enzymes (termed iridoid synthases; ISY) are involved in iridoid biosynthesis (Geu-Flores et al. 2012), and Munkert et al. (2015a, b) reported that iridoid synthase activity is common among the plant progesterone 5β-reductase family. More recently, the enzymes progesterone 5β-reductase/iridoid synthase-like enzymes were termed PRISEs (Schmidt et al. 2018) and described as a catalytic reservoir for specialized metabolism across land plants (Nguyen and O’Connor 2020). Progesterone in cardenolide biosynthesis and 8-oxogeranial in iridoid synthesis are not the only substrates for PRISEs. Various 1,4-enones, such as 2-cyclohexen-1-one, methyl vinyl ketone or citral are also accepted (e.g.,Durchschein et al. 2012; Munkert et al., 2015a, b). PRISEs seem to be involved in reactions in central plant metabolism and appeared early in plant evolution, presumably with lateral gene transfer originating from α-proteobacteria (Tarrío et al. 2011).

Wounding or environmental stressors such as high salt concentrations or low temperatures enhanced *A. thaliana St5βR* (At4g24220, termed AWI 31 by Yang et al. 1997) expression (Winter et al. 2007). *A. thaliana* developed a phenotype with altered vein patterning, when At4g24220 (a PRISE gene termed *VEP1* by Jun et al. 2002) was knocked out. The first *P5βR* gene described, named *P5βR1* (Herl et al., 2006) in *Digitalis lanata* seemed to be expressed constitutively at a much higher level than *P5βR2* (Perez-Bermúdez et al. 2010). On the other hand, *P5βR2* expression is related to stress not only in *Digitalis* but in other cardenolide-containing plants like *Erysimum* as well (Perez-Bermúdez et al. 2010; Horn et al. 2020).

As far as engineering of the cardenolide formation is concerned, Sales et al. (2007) transformed *D. minor* plants with an N-truncated form of a *3-hydroxy-3-methylglutarate CoA reductase* gene whose constitutive expression resulted in increased accumulation of sterols and cardenolides. More recently, Kairuz et al. (2020) reported the introduction of an *A. thaliana* PRISE into *D. purpurea*, but could not demonstrate increased cardenolide accumulation indicating that progesterone 5β-reduction is not the rate-limiting step in cardenolide biosynthesis.

The connection between *progesterone 5β-reductase* gene expression, plant stress response and cardenolide formation was investigated here by generating RNAi-mediated knockdown of *DlP5βR1* (AY574950.1) and *DlP5βR2* (HM210089.1) in shoot cultures of *D. lanata*. These shoot cultures provided stable, homogeneous and reliable systems to study the effects of exogenous compounds on gene expression and cardenolide formation. To eliminate the possibility that effects attributed to gene knockdown may have been provoked by stress, we investigated the influence of glutathione (GSH), buthionine sulfoximine (BSO), methyl vinyl ketone (MVK), and progesterone on cardenolide formation and *progesterone 5β-reductase* gene expression in wild-type and knockdown shoots. Glutathione strongly inhibited cardenolide biosynthesis in embryogenic cell cultures of *D. lanata* (Berglund and Ohlsson 1993) whereas buthionine sulfoximine, an inhibitor of glutathione biosynthesis, reversed this glutathione effect (Berglund and Ohlsson, 1993). Methyl vinyl ketone (MVK), a reactive electrophile species (RES) and a stress metabolite (Kai et al. 2012), is also known to induce a plant stress response (Alméras et al. 2003). Progesterone, a cardenolide precursor, could be regarded as a reactive electrophile species (RES) which can be detoxified by PRISEs. We, therefore, analyzed the effect of both progesterone and MVK on plants harboring an RNAi-mediated knockdown of *DlP5βR*. Finally, we here demonstrate that *P5βR1* is directly involved in constitutive cardenolide biosynthesis. On the other hand, *P5βR2* seems to be more important for immediate but transient cardenolide formation in stress response situations.

## Materials and methods

### *Agrobacterium tumefaciens*-meditated genetic transformation

The genetic transformation protocol commenced using axenic shoot cultures from *D. lanata* (line *Dl*1681*)* grown on solid MS medium (Murashige and Skoog 1962). Shoots intended for genetic transformation were transferred and sub-cultivated in half-strength liquid MS medium containing per liter: 35 g sucrose, 1.0 mg 6-benzylaminopurine (BAP) and 0.1 mg indole-3-acetic acid (IAA). The cultures were kept on gyratory shakers (80 rpm, 24 °C) in permanent light (~ 40 μmol photons m^−2^ s ^−1^) and sub-cultivated as advised by Stuhlemmer et al. (1993).

Leaflets were excised and transferred onto solid MS medium containing 1.5 mg L^−1^ thidiazuron (TDZ) and 0.1 mg L^−1^ NAA (medium I). After 2 days, leaf explants were first infiltrated under vacuum for 30 s and then bathed in a suspension of *Agrobacterium tumefaciens* strain GV3101 containing either *GUS* and *nptII* gene on 679p935s-GusIo-rbs vector or RNAi fragments and *nptII* gene on pHellsgate8 vector (oD_600_ = 0.6 – 0.8; acetosyringone, 0.3 mg mL^−1^) for 30 min and transferred back onto medium I. For verification of the transformation system, we used the construct 679p935s-GusIo-rbs (provided by the Institute of Horticultural Production Systems, Section Floriculture, Gottfried-Wilhelm-Leibniz-Universität Hannover, Germany), containing a *nptII* gene mediating kanamycin resistance and a *GUS* reporter gene. Both genes were under the control of CaMV 35S promoters, for permanent gene expression. Leaf explants and adhering bacteria were co-cultivated for 2 days solid on medium I. Leaf explants were then transferred onto solid medium II containing 100 mg L^−1^ kanamycin and 250 mg L^−1^ cefotaxime for selection (medium II). Medium II was exchanged weekly until sturdy shoots developed after 4–6 weeks. These were transferred onto solid MS medium containing 30 g L^−1^ sucrose and 2.25 mg L^−1^ BAP (DDV medium) supplemented with 150 mg L^−1^ kanamycin and 250 mg L^−1^ cefotaxime (medium III). Shoots were kept under these conditions until further use with medium III exchanged weekly. To exclude the presence of the Ti-plasmids in transgenic shoots, gDNA was checked by PCR reaction against *spectinomycin* (*SmR*) and *virD2* gene (Suppl. Table S1; Suppl. Fig. S1).

#### Identification of transgenic shoots

Genomic DNA (gDNA) was isolated from *D. lanata* shoots according to Allen et al. (2006). A maximum of 100 mg plant material was ground in cold extraction buffer in a 1.5 mL reaction tube using a small pestle. The solution was rapidly heated to 65 °C and kept under these conditions for 30 min. The gDNA pellets obtained by centrifugation (20,000 × *g*; 20 °C) were dried at 30 °C and resuspended in sterile water.

Integration of T-DNA was verified by PCR with gDNA from shoots grown on medium I and primers against the genes *nptII* and *GUS* inserted in the T-DNA region. Additional PCRs against *spectinomycin* (*SmR*) resistance gene and *virD2* gene were conducted to exclude the presence of Ti-plasmids (Suppl. Table S1; Suppl. Fig. S1). For PCR, FastGene Optima HotStart Ready Mix (Nippon Genetics Europe GmbH, Düren, Germany) and FlexCycler^2^ (Analytik Jena AG, Jena, Germany) were used. The PCR program consisted of an initial denaturation (95 °C for 60 s), 29 cycles (95 °C for 15 s; T_A_ (Suppl. Table S1) for 30 s; 68 °C for 30 s) and a final amplification (68 °C for 600 s).

GUS activity was demonstrated by histochemical staining (Jefferson et al. 1987).

#### RNAi-mediated knockdown of *P5βR1* and *P5βR2* in *Digitalis lanata* L. shoots

For downregulating *P5βR1* and *P5βR2 gene* expression, RNA interference (RNAi) technology and the plant binary pHellsgate8 vector system was used. Small specific fragments of around 230 bp of *P5βR1* and *P5βR2* were identified by sequence alignment. The Gateway cloning technique was used to yield the final vector constructs (Suppl. Fig. S2). Therefore, the specific fragments of *P5βR1* and *P5βR2* were amplified using the following primer pairs flanked by attB sites for homologues recombination.

P5βR1 for: GGGGACAAGTTTGTACAAAAAAGCAGGCTTCATGAGCTGGTGGGCTG.

P5βR1 rev: GGGGACTTTGTACAAGAAAGCTGGGTCTTAATTGATCGGATTATCCTCA.

P5βR2 for: GGGGACAAGTTTGTACAAAAAGCAGGCTTCATGTATACCGACACAACGACTTGG;

P5βR2 rev: GGGGACCACTTTGTACAAGAAAGCTGGGTCTTGTCGGAAAGTGGAGACAATT.

The resulting PCR products were cloned into pDONR^®^221 using BP-Clonase^®^ enzyme mix (Thermo Fisher Scientific Inc., Waltham, USA). These plasmids were used as entry vectors for the Gateway cloning system mix (Thermo Fisher Scientific Inc., Waltham, USA) into pHellsgate8 (Division of Plant Physiology, University Bayreuth; Suppl. Fig. S2). The resulting plasmids were checked by sequencing (Eurofins, Ebersberg, Germany). Chemical competent *A. tumefaciens* strain GV3101 cells (100 µL of cell suspension) were transformed with 1 µg of each plasmid by three sequential incubation steps of 5 min each on ice, liquid nitrogen, and at 37 °C. After the addition of 750 µL SOC-medium, the cells were incubated for 4 h at 28 °C and afterward spread on solid LB medium containing 10 µg mL^−1^ rifampicin and 50 µg mL^−1^ kanamycin. Individual cell clones were checked by colony PCR and those found positive were used for the genetic transformation of *D. lanata* shoots.

### Quantitative real-time analysis (qPCR)

*D. lanata glutathione reductase* (*GR*) and *glutathione S-transferase* (*GST*) genes were identified using the transcriptomic search tool of medicinal plant genome (http://medicinalplantgenomics.msu.edu/; Suppl. Table S2). Primers for amplifying *P5βR1* and *P5βR2* were identified using *D. lanata P5βR1* (AY574950.1) and *P5βR2* (HM210089.1) GenBank entries. The primers used did not bind to the regions used for RNAi-mediated knockdown. Primer pairs amplifying a *D. lanata actin* reference gene sequence were used as described by Ernst et al. 2010 (Suppl. Table S2).

To analyze the transcription rates, total RNA was isolated from shoots cultivated in vitro using Monarch^®^ Total RNA Miniprep Kit (New England Biolabs, Ipswich, USA). cDNA was synthesized using 500 ng total RNA employing the RevertAid H Minus First Strand cDNA Synthesis Kit (Thermo Fisher Scientific Inc., Waltham, USA). For qPCR, samples were prepared using the FAST SYBR Green Mastermix Kit (Applied Biosystems, Germany) according to the user manual. qPCR was carried out with the StepOnePlus Real-Time PCR System (Life Technologies, Germany) and the relative gene expression levels were calculated using the 2^−ΔΔCt^ method (Schmittgen and Livak 2008) with *actin* as the reference gene.

### Quantification of 5β-cardenolides

Cardenolides were extracted as described by Wiegrebe and Wichtl (1993). Extracted cardenolides were hydrolyzed according to Schaller and Kreis (2006) to enhance sensitivity. The cardenolide aglyca released (digitoxigenin and digoxigenin) were detected and quantified by UPLC at 220 nm: digitoxigenin (RT = 4.9 min) and digoxigenin (RT = 2.9 min). Progesterone was used as the internal standard (RT = 6.8 min). Analysis was carried out in an ACQUITY Ultra Performance LC™ system (Waters, Milford, MA, USA) linked to TUV detector (Waters, Milford, MA, USA). 5–10 µL of sample solution diluted with acetonitrile 1:5 was injected into a reversed phase column (Phase: 1.7 µm Fortis C18, column dimension: 50 × 2.1 mm, Fortis Technologies Ltd, Cheshire, England, UK) kept at 40 °C. The mobile phase consisted of solvent A (H_2_O/0.1% HCOOH) and solvent B (acetonitrile/0.1% HCOOH). The flow-rate was 0.4 mL min^−1^; *t* = 0 min, 5% B; *t* = 10 min, 95% B; *t* = 11 min, 5% B; *t* = 13 min, 5% B.

### Measuring progesterone 5β-reductase activity

The progesterone 5β-reductase activity assay contained: 1 mg mL^−1^ of the respective crude protein extract, 0.3 mM progesterone and a NADPH regenerating system consisting of 1.1 mM glucose 6-phosphate, 6.4 mM NADP^+^ and 4.2 nkat glucose-6-phosphate dehydrogenase. Assays were stopped after 4 h by adding 1 mL CH_2_Cl_2_. Heat inactivated samples (10 min 99 °C in thermoblock) served as control for no substrate conversion. Consumption of the substrate was determined by GC–MS as described by Ernst et al. (2010). A Shimadzu GC2010/QP-2010S was used in IE mode with helium as the carrier gas (flow: 1.2 mL min^−1^). A DB-5 ms column (J&W GC column) from Agilent (30 m × 0.25 mm × 0.25 μm) was used. The program commenced at 200 °C for 4 min followed by a 20 °C min^−1^ linear increase up to 290 °C and finished with 4 min at 300 °C. Authentic standards were from Steraloids Inc. (Newport, RI, USA).

### Quantification of progesterone

The preparation of plant extracts followed the extraction method of Iino et al. (2007) with slight modifications. 5 g *D. lanata* shoots were frozen in liquid nitrogen, ground to a fine powder and extracted with 50 mL MeOH at room temperature (23 °C). The filtrate was evaporated and the residue dissolved in 50 mL EtOAc and washed twice with 50 mL 0.5 M K_2_HPO_4_. The organic phase was evaporated, the residue dissolved in 20 mL hexane and extracted twice with 20 mL 20% MeOH. The combined MeOH phases were diluted with 40 mL H_2_O and extracted twice with 60 mL hexane each. The combined hexane phases were evaporated, the residue dissolved in CH_2_Cl_2_ and filtered through 5 g activated carbon. The filtrate was evaporated again and the residue analyzed further. Progesterone was identified by GC–MS using above mentioned GC–MS program. The fidelity of progesterone detection was further verified by using samples spiked with 100 µmol progesterone. The retention time of the progesterone peak was at 10.15 min and mass spectrometric analysis indicated the separation of progesterone ions at m/z 314, 272, 124 and 43. Progesterone was quantified by UPLC (at 220 nm). The solvent gradient was set as follows: solvent A (H_2_O): 95%, solvent B (acetonitrile): 5%, flow-rate: 0.4 mL min^−1^ by *t* = 0 min, 5% B; *t* = 10 min, 95% B; *t* = 11 min, 5% B; *t* = 13 min, 5% B.

### Estimation of glutathione pool

Total glutathione (t-GSH) and oxidized glutathione (GSSG) were measured according to Hajdinak et al. (2018). Fresh *D. lanata* shoots were homogenized with 6% metaphosphoric acid (w/v, containing 1 mM EDTA) and centrifuged at 16,000×*g* for 15 min at 4 °C (Sahoo et al. 2017). Removing reduced GSH for measuring oxidized glutathione (GSSG) followed the procedure described by Rahman et al. (2006). To assay t-GSH and GSSG, 85.2 µL of 50 mM sodium phosphate buffered (pH 7.5), 3 µL nicotinamide adenine dinucleotide phosphate (NADPH, 0.3 mM) and 2 µL of each supernatant were pipetted into a microtiter plate. The reaction was started by adding 5.3 µL of yeast glutathione reductase (GR, 1.6 U/ mL). The plate was incubated for 15 min at 25 °C. Finally, 4.5 µL 2-nitrobenzoic acid (DTNB, 0.225 mM) was added and the change of absorption measured spectrophotometrically at 405 nm against a blank. Results were expressed as nmol per gram fresh weight (nmol * g^−1^ FW). GSH content was calculated by subtracting GSSG from total glutathione (Rahman et al. 2006).

### Treatment with α,β-unsaturated carbonyls and 5β-pregnane-3,20-dione

WT shoots in liquid medium were fed with progesterone (PO; 0.16 mM final concentration in DMSO) to analyze the effects of progesterone, a precursor of 5β-cardenolide formation. 5β-Cardenolides were quantified by UPLC 1, 2, 3, 7 and 8 days after progesterone treatment. Expressions of the *P5βR1* and *P5βR2* genes were quantified by qPCR using *actin* as the reference gene.

WT and transgenic shoots were treated with progesterone (PO) and 5β-pregnane-3,20-dione (PR; both 0.3 mM final concentration in DMSO) and after 7 days of treatment cardenolide levels were quantified as described above.

To analyze the effects of other α,β-unsaturated carbonyls on progesterone 5β-reductase expression and cardenolide content, WT and transgenic shoots treated with 2 µmol L^−1^ air volume MVK (final concentration; Merck KGaA, Darmstadt, Germany) diluted in tab water for 3 h as described by Munkert et al. (2014). Water served as the control. Expressions of *P5βR1*, *P5βR2 and GST* were quantified by qPCR and cardenolide content analyzed as described above.

### Glutathione (GSH) and buthionine sulfoximine (BSO) treatment

Transgenic shoots were transferred to medium III and WT shoots to DDV containing 0.3 mM BSO or 0.3 mM GSH as described by Berglund and Ohlsen (1993). In addition, WT shoots were treated with a combination of 0.04 mM BSO and 0.3 mM GSH as well as 0.3 mM BSO and 2 mM GSH as suggested by Berglund and Ohlsen (1993).

### Heterologous expression of *Dl*P5βR1 and *Dl*P5βR2

Primer pairs for restriction-free cloning of *Dl*P5βR2 into expression vector pDEST17 were designed using rf-cloning.org (Bond and Naus 2012).

Forward primer: 5′GCTCGAATACCCCAGAACATATGAGYTGGTGGTGGGCT′3, reverse primer: 5′TTGTACAAGAAATCTGGGTCTCAAGGAACAATCTTGTAAGC′3. The cloning procedure was realized as described by Unger et al. (2010). Amplification of “megaprimers” and cloning PCR were conducted in a FlexCycler^2^ thermocycler (Analytik Jena, Jena, Germany) using Phusion^®^ High-Fidelity DNA Polymerase (New England Biolabs, Ipswich, USA). Cloning products were used for the transformation of *E. coli* strain DH5α. Colonies showing resistance against ampicillin were tested by colony PCR using *Taq* DNA Polymerase (New England Biolabs, Ipswich, USA). Plasmids of positive colonies were isolated using Roti^®^-Prep Plasmid MINI (Carl Roth GmbH, Karlsruhe, Germany). The correct integration of *Dl*P5βR2 into the expression vector was verified by sequencing (Eurofins Genomics, Ebersberg, Germany). Standard primers against T7 promotor or against T7 terminator were used. *E. coli* SoluBL21 were transformed with positive constructs. Positively identified *E. coli* SoluBL21 colonies were used for heterologous expression of *Dl*P5βR2.

For heterologous expression of *Dl*P5βR1, a pQE30 vector (Herl et al. 2006) was used. For expression, bacteria were cultivated in 1 L LB medium containing ampicillin or ampicillin and kanamycin (pDEST17 vector: 100 mg mL^−1^; LB_Amp_; pQE30 vector: 100 mg mL^−1^; 25 mg mL^−1^; LB_Amp;Kan_) until an optical density of 0.5–0.7 was reached. IPTG was added to a final concentration of 0.1 mM to induce heterologous gene expression. Bacteria were cultivated at 4 °C for 96 h. Low temperatures reduced the formation of “inclusion bodies” and enhanced the amount of soluble protein (Stevens 2000) and was successfully used for the expression of P5βRs by Herl et al. (2006) and several other P5βR from different plant species (Munkert et al. 2015a, b). Cells were harvested by centrifugation (4000×*g*, 20 min, 4 °C) and the pellet was either used directly for protein isolation or stored at −20 °C for further use.

Pellets were thawed on ice and resuspended in 10 mL lysing buffer (50 mM NaH_2_PO_4_, 300 mM NaCl, 40 mM imidazole). 20,000 U mL^−1^ lysozyme (Carl Roth GmbH, Karlsruhe, Germany) was added and the solution incubated under shaking (30 min, 100 rpm). Subsequently cells were disrupted by ultrasonication (6 times for 30 s) and centrifuged (10,000×*g*, 40 min, 4 °C).

The supernatant was used for the purification of recombinant proteins by immobilized metal chelate affinity chromatography (IMAC). HisTrap™ HP columns (1 mL) and ÄKTA purifier chromatography system was used (GE Healthcare, Uppsala/Sweden). Protein concentration was determined according to Bradford (1976).

Proteins were separated by SDS-PAGE and semi-dry immunoblotting was carried out according to the QIAexpress Detection and Assay Handbooks (QIAgen) with slight modifications as described by Munkert et al. (2015a, b). Recombinant proteins were separated on a 12% Bis–Tris polyacrylamide gel and then transferred onto a nitrocellulose membrane by electroblotting. After blocking with 5% milk powder in Tris-buffered saline with 0.1% Tween 20, the membrane was incubated for 1 h with mouse anti-His antibodies (mixture of RGS-, Tetra-, and Penta-His antibodies; dilution 1:2000; QIAgen, Hilden, Germany). Anti-mouse IgG-peroxidase antibody (Sigma, Munich, Germany) was used as the detection antibody (dilution 1:10,000; incubation 1 h). Chemiluminescence of 3-aminophthalate released from luminol was used for detection.

#### Determination of kinetic constants of heterologous *Dl*P5βR1 and *Dl*P5βR2

To determine the kinetic constants of recombinant P5βR proteins, reductase activity was measured spectrophotometrically, as described before (Burda et al. 2009; Bauer et al. 2010; Munkert et al. 2015a, b). The conversion of NADPH/H^+^ (0.2 mM) (AppliChem GmbH) to NADP^+^ was monitored at 340 nm in the presence of the respective substrates over a time course of 5 min at 40 °C. Depending on the P5βR investigated, between 0.01 and 0.1 mg × mL^−1^ of recombinant protein, 0.2 mM NADPH/H^+^, and varying concentrations of the substrate (0.01–1.6 mM) were used in the assay. Assays without the respective substrate were used as control.

#### Statistical analysis

All data are expressed as the mean ± SEM. Each individual experiment was composed of n ≥ 3 biological and technical replicates. Means between the various groups were compared by one-way analysis of variance (ANOVA followed by Tukeyʼs post hoc test). In case of multiple comparisons, a post hoc Bonferroni correction was applied. *P* values < 0.01 and < 0.05 were considered statistically significant. Data were analyzed using GraphPad Prism 5 Software (GraphPad).

## Results and discussion

### Characterization of recombinant r*Dl*P5βR1 and r*Dl*P5βR2

Previously, various PRISEs of *Digitalis* and other plants have been heterologously expressed in *E. coli* and yeast and enzyme kinetics have been studied (Herl et al., 2006; Munkert et al. 2015a, b; Rieck et al. 2019). All available results (e.g., Herl et al. 2006; Perez-Bermúdez et al. 2010; Munkert et al. 2015a, b) show that PRISE-encoding gene *P5βR1* is expressed constitutively in plants. In contrast, *P5βR2* gene expression increased immediately after wounding or exposure to chemical stress resulting in elevated cardenolide levels in *D. purpurea*. Since recombinant *Dp*P5βR2 has a higher substrate affinity for progesterone than recombinant *Dp*P5βR1, it was suggested that *Dp*P5βR2 is involved preferentially in cardenolide biosynthesis (Perez-Bermúdez et al. 2010). We here used the *DpP5βR*2 sequence described in the above publication to deduce primers allowing for the isolation and heterologous expression of a *D. lanata* homologue, termed *DlP5βR*2 (Suppl. Fig. S3). Recombinant forms of *Dl*P5βR1 and *Dl*P5βR2 (termed r*Dl*P5βR1 and r*Dl*P5βR2) were characterized with regard to their kinetic data for progesterone and its small mimic MVK (Table 1). r*Dl*P5βR2 converted progesterone much faster than r*Dl*P5βR1. On the other hand, r*Dl*P5βR1 clearly discriminated between progesterone and MVK whereas r*Dl*P5βR2 did not. MVK was accepted much better by r*Dl*P5βR1 than by r*Dl*P5βR2 indicating that *Dl*P5βR1 and *Dl*P5βR2 may play different roles within the plant. Differences in substrate specificity were similarly observed in other orthologue PRISEs in different plant species, such as *Medicago, Catharanthus, Erysimum*, and *Arabidopsis* (Munkert et al. 2015a, b; Nguyen and O’Connor 2020).

**Table 1:** Kinetic constants of r*Dl*P5βR1 and r*Dl*P5βR2 for progesterone and methyl vinyl ketone (MVK)

	K_M_ [mM]	k_cat_ [s^−1^]	k_cat_/K_M_ [M^−1^/s^−1^]	k_cat_/K_M_ MVK:k_cat_/K_M_ progesterone
r*Dl*P5βR1				
Progesterone^a^	0.36	0.02	52.2	97
MVK	0.27	1.37	5074	
r*Dl*P5βR2				
Progesterone	0.23	0.48	2115	0.56
MVK	0.21	0.26	1189	

^a^Data from Bauer et al. (2012)

As both known PRISEs from *D. lanata* differ in their substrate specificity, one may assume different functions *in planta*. To address this question, we here generated RNAi-mediated gene knockdowns of *DlP5βR*1 and *DlP5βR*2 in *D. lanata.*

### Stable transformation of *Digitalis lanata* L. shoots

**Fig. 2:**
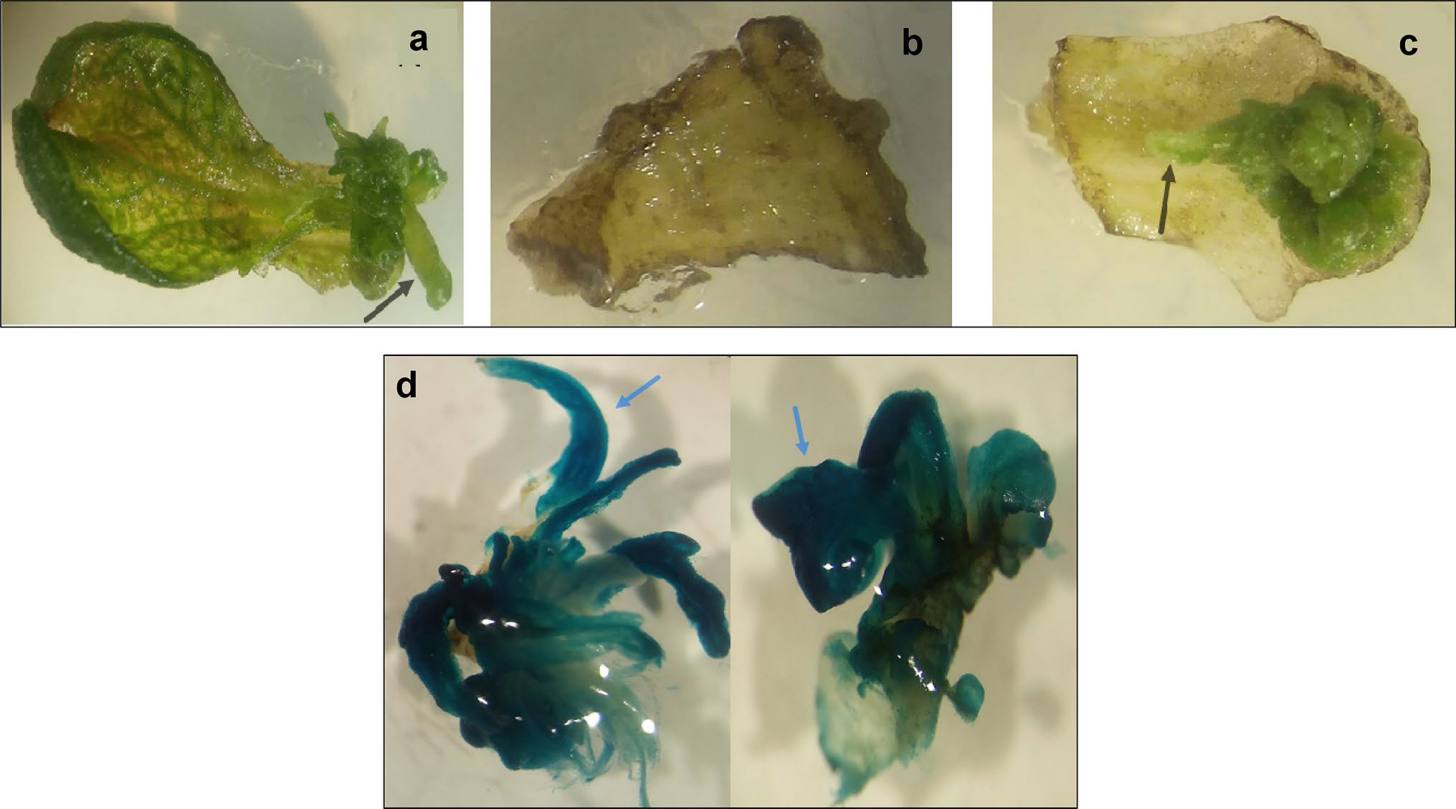
Development of shoot tissue on leaf explants after *Agrobacterium* transformation. **a** Positive control, **b** negative control, and **c** transformed explant. Shoots developed in the positive control and the transformed explant (black arrows) while the negative control showed only small areas with callus formation. **d** Histochemical GUS staining was used to detect the expression of the introduced *GUS* gene. Blue arrows show parts of the stained areas

Shoots of the *Digitalis lanata* shoot culture line *Dl*1681 (*Dl* WT) were used for genetic transformation to ensure homogeneity in each line. Shoots were kept in liquid medium for approximately 9 days where they altered their morphology and became more susceptible to infection with *Agrobacterium tumefaciens.* The *A. tumefaciens* strain GV3101 containing the vector 679p935s-GusIo-rbs was used in the control transformation experiments (*Dl* VC). After 2 days of co-cultivation on solid medium I, leaf explants were transferred to selection medium II containing kanamycin and cefotaxime. Suitable selection conditions were verified by a negative control (explants transformed with *Agrobacterium tumefaciens* not carrying the *nptII* and *GUS* genes) and a positive control (non-transformed explants). Figure 2 shows a positive control on medium I (a), negative control after 3 weeks cultivation on medium II (b) and a transformed explant (c).

Shoot tufts developing on transformed explants were transferred onto medium III and cultivated further. Stable integration of *GUS* and *nptII* was verified by PCR (Suppl. Fig. S1). GUS expression was also demonstrated histochemically (Fig. 2d). To ensure the absence of the *Ti*-plasmid, PCR against *virD2* and the spectinomycin-resistance gene (*spec*) was carried out (Suppl. Fig. S1). About 5% of explants subjected to the transformation protocol established here developed into transgenic shoot tufts.

*Digitalis* comprises several important medicinal plant species, producing biologically active 5β-cardenolides. Therefore, a plenitude of protocols for conducting biochemical and molecular biological experiments have been established (Luckner and Wichtl 2000; Kreis 2017). Several groups reported the generation of genetically modified tissues (Moldenhauer et al. 1990; Pinkwart et al. 1998) and plants (Saito et al. 1990; Lehmann et al. 1995; Thomar et al. 1998; Sales et al. 2007; Pérez-Alonso et al. 2014; Kairuz et al. 2020) using *Agrobacterium tumefaciens*-mediated genetic transformation of protoplasts, embryo-like cell clusters, or leaf discs. Pradel et al. (1997) used *A. rhizogenes* to induce hairy root formation in *D. lanata* and regenerated transgenic plants. Regenerating intact plants, using any of these approaches, proved to be rather inefficient. For example, Lehmann et al. (1995) described that even in the best case scenario only 5% of all leaf explants produced callus and induction of organ development was not successful. Shoots regenerated from transformed protoplast showed an exceptionally low transformation rate (Lehmann et al. 1995). We here adopted permanent shoot cultures as the starting material to establish a reliable transformation protocol using *A. tumefaciens*-mediated genetic transformation (Barton et al. 1983). Our protocol yielded approximately 5% transformed shoots which was similar to the recently reported rates for *Digitalis purpurea* (Kairuz et al. 2020).

### RNAi-mediated gene knockdown of *DlP5βR*1 and *DlP5βR2* in *Digitalis lanata* shoots

The shoots used for *Agrobacterium*-mediated transformation were propagated in vitro. The transformation protocol described above was used for RNAi-mediated gene knockdown of *DlP5βR1* (described by Herl et al. 2006) and *DlP5βR2*. *DlP5βR2* is a homologue of *D. purpurea P5βR2,* first reported by Pérez-Bermúdez et al. (2010) when investigating the effect of various kinds of stress in *D. purpurea*. They found *DlP5βR2* to be only weakly expressed in various tissues of *D. purpurea* but induced immediately after stress. This was paralleled by a transient boost in cardenolide formation. Homology-based screening in the available gene databases indicated the presence of more than two progesterone reductases in *D. purpurea* and *D. lanata* (Schmidt et al. 2018). We here focused on the two known genes only, accepting that the knockdown of them might not silence progesterone reductase activity completely. The transgenic shoot cultures established here proved to be an excellent system to address the questions related to the physiological impact of *P5βR1* and *P5βR2* in *D. lanata.* The transformed shoots remained unchanged for an extended period of time (over 3 years), produced cardenolides and allowed for experiments in a defined environment.

Gateway cloning employing the pHellsgate8 vector yielded several *P5βR* knockdown lines of *D. lanata* shoots. Constructs containing RNAi fragments designed against *DlP5βR1* or *DlP5βR2* were used for the transformation experiments*.* Expression of *nptll* was demonstrated by PCR to verify genetic transformation and integration of T-DNA into the plant genome (Suppl. Fig. S1).

**Fig. 3:**
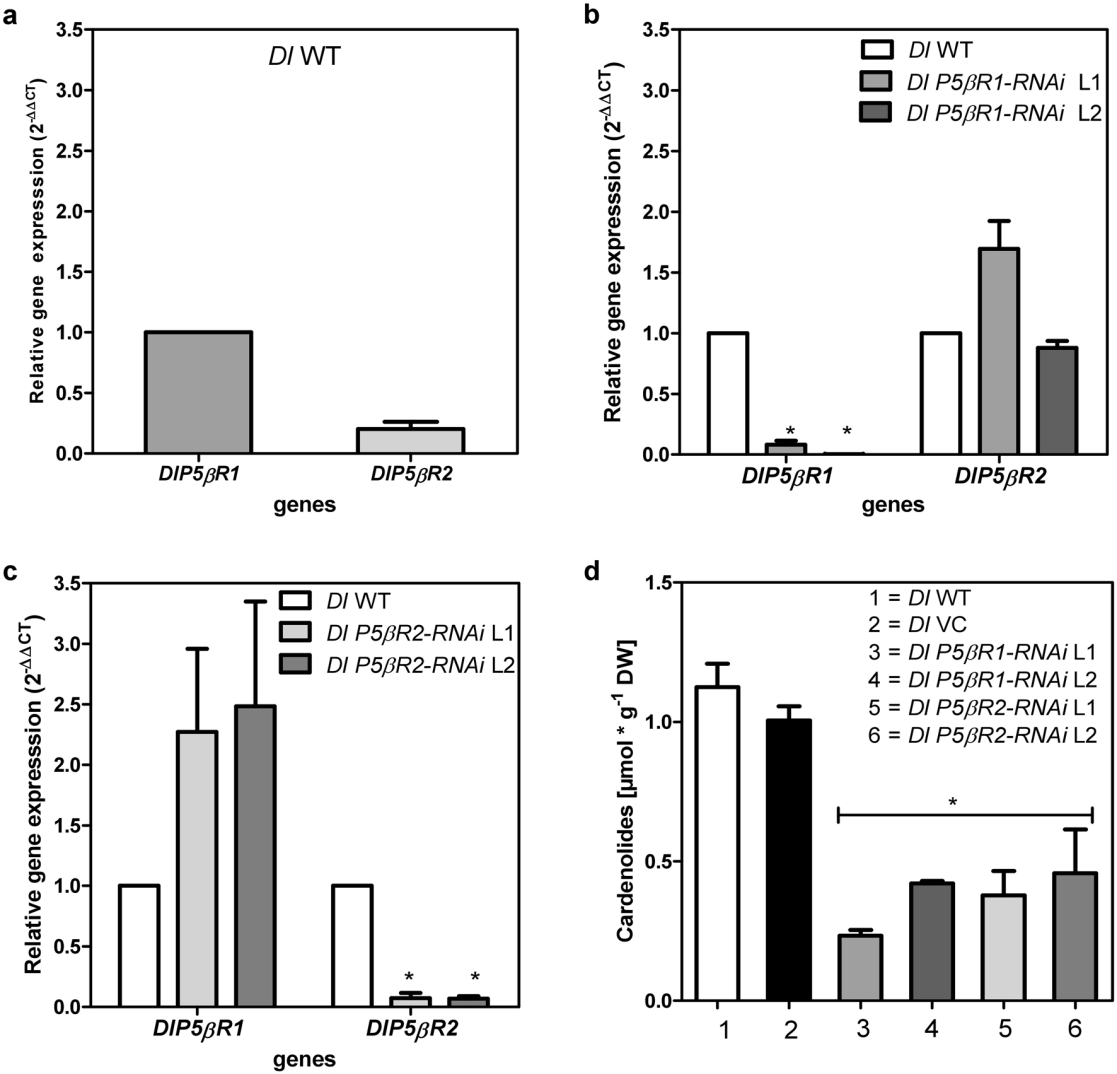
Gene expression of *DlP5βR1* and *DlP5βR2 in Digitalis lanata WT and* RNAi-mediated knockdown shoots. Relative RNA expression levels were calculated using the qPCR method by applying the 2^−ΔΔCT^ method with actin as the reference gene. The y axis denotes the normalized relative transcript accumulation of the genes indicated in the x axis (mean ± SEM, *n* = 3; Tukey’s test, *P > 0.01). (**a**) Relative transcripts of *P5βRs* (*DlP5βR1* and *DlP5βR2*) in *D. lanata* WT; (**b**) relative expression of *DlP5βR1* and *DlP5βR2* in a *Dl P5βR1*-*RNAi* knockdown line 1 (L1) and line 2 (L2) compared to WT shoots; **c** relative expression of *DlP5βR1* and *DlP5βR2* in a *Dl P5βR2-RNAi* knock down line 1 (L1) and line 2 (L2) compared to WT shoots; (d) quantification of digoxigenin and digitoxigenin in *D. lanata* WT shoots and transgenic shoots with reduced expression of *P5βR*s. Shoots transformed with 679p935s-GusIo-rbs (VC) were used as control to exclude artificial effects created by the transformation process (mean ± SEM; *n* = 9; Tukey’s test, *P > 0.05)

The expression of *P5βR1* or *P5βR2* in wild-type (WT) shoots as well as in RNAi-mediated gene knockdown lines was evaluated by qPCR (Fig. 3). Relative expression of *P5βR1* in *D. lanata* WT shoots was around five times higher than that of *P5βR2* (Fig. 3a)*.*

To demonstrate that the *Agrobacterium* transformation system itself does not influence P5βR expression, *D. lanata* shoots were transformed with 679p935s-GusIo-rbs (vector control) not harboring the RNAi constructs. qPCR revealed that *P5βR* expression was not influenced by the genetic transformation method used (Suppl. Fig. S4). On the other hand, all RNAi knockdown lines showed a strong reduction of either *P5βR1* or *P5βR2* expression. Exemplary gene expression is shown in two RNAi-mediated gene knockdown lines (Fig. 3b, c). In *Dl P5βR1*-*RNAi* knockdown lines *P5βR1* expression was only 8–10% of that in WT shoots. *P5βR2* expression in *Dl P5βR2*-*RNAi* knockdown lines was 7–13% of that in WT shoots. Knockdown of *DlP5βR1* only slightly affected the expression of *DlP5βR2* and vice versa (Fig. 3b, c). Gene-specific knockdown of the respective *P5βR* remained stable in the *P5βR-RNAi* lines used here for over 3 years in defined conditions.

P5βR protein activity was considerably lower in *P5βR*-*RNAi* knockdown lines than in WT shoots. Compared to heat inactivated controls WT shoots and the vector controls were able to convert 30–34%, of the fed progesterone, whereas *P5βR-RNAi* lines converted only 0–10%. This was indicative of the successful knockdown of the respective *P5βR* genes.

The influence of *P5βR* expression on cardenolide formation in *D. lanata* shoot cultures was investigated in some detail. Cardenolides were extracted in the central part of a culture cycle from WT shoots, from shoots transformed with the 679p935s-GusIo-rbs vector (vector control termed *Dl* VC) and from all *Dl P5βR*-*RNAi* knockdown lines. Digitoxigenin and digoxigenin, released after acidic hydrolysis of the cardenolides present in the extracts, were quantified by UPLC. Cardenolide levels of around 1 µmol g^−1^ DW were determined in WT shoots and vector controls, indicating that the transformation method had not influenced cardenolide levels. Cardenolide levels of WT shoots were very similar to those of earlier studies of *D. lanata* shoot cultures (Stuhlemmer et al. 1993; Christmann et al. 1993; Eisenbeiß et al. 1999) who reported levels of 0.6–1.0 µmol g^−1^ DW. Cardenolide contents were much lower in all *Dl P5βR*-*RNAi* knockdown lines. Cardenolide levels of 0.23–0.42 µmol g^−1^ DW and 0.38–0.46 µmol g^−1^ DW were detected in the *Dl P5βR1-RNAi* and *Dl P5βR2-RNAi* lines, respectively (Fig. 3d; exemplary for two *Dl P5βR*-*RNAi* knockdown lines) indicating that the lack of either of the PRISEs investigated here can impair cardenolide formation. The higher efficiency of rP5βR2 for progesterone conversion (Table 1) can maybe compensate for its lower expression abundancy (Fig. 3a). This may be taken as the first direct proof that P5βRs are involved in cardenolide biosynthesis as assumed previously (Gärtner et al. 1994; Kreis 2017).

### Effects of glutathione and buthionine sulfoximine on *P5βR* gene expression as well as on cardenolide content

**Fig. 4:**
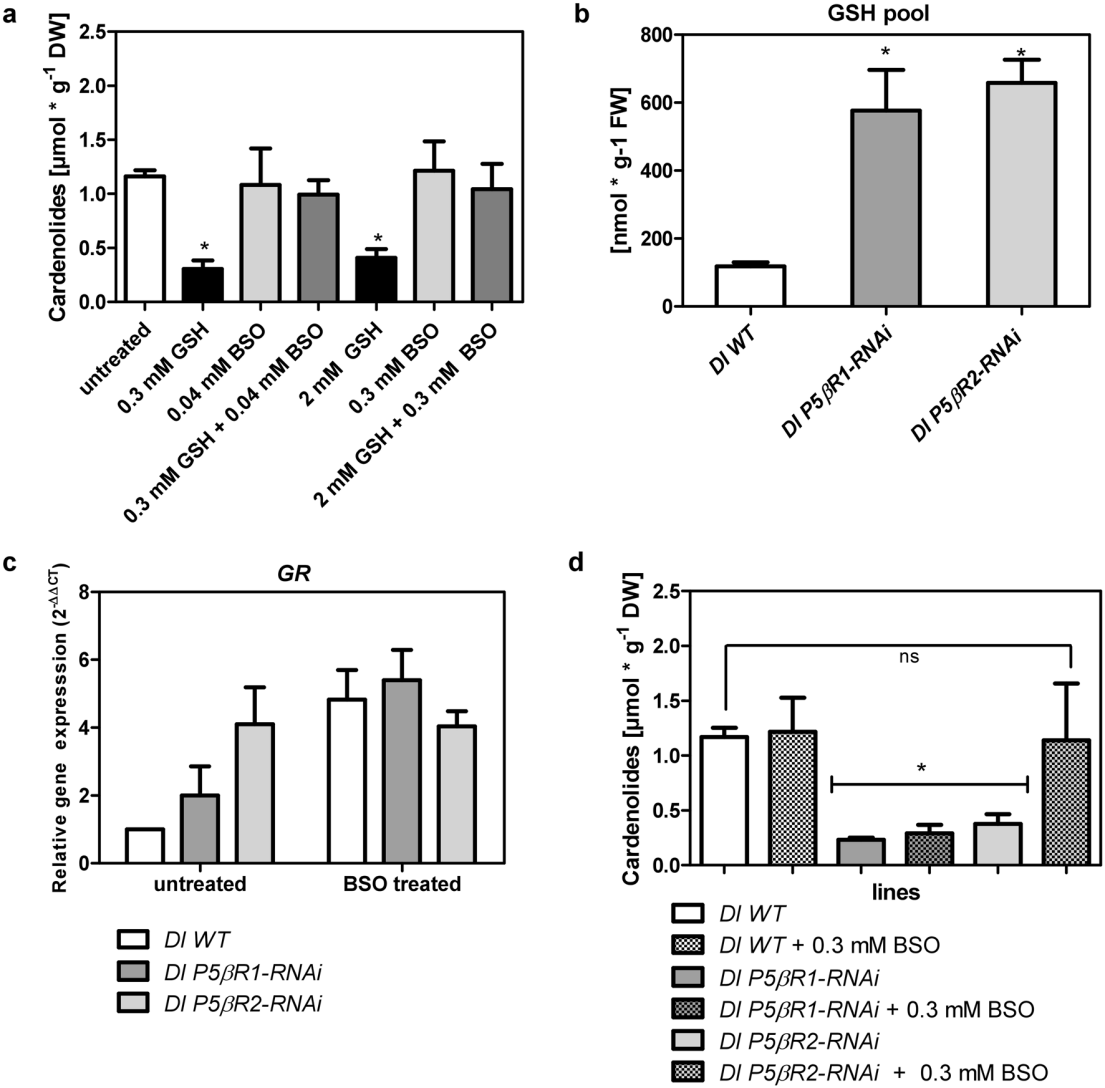
Effects of glutathione (GSH) and buthionine sulfoximine (BSO) treatment. **a** Effects of GSH and BSO alone or a combination on cardenolide level in *D. lanata* WT shoots; **b** estimated GSH pool in *Dl* WT and *Dl P5βR-RNAi* knockdown lines calculated by measuring t-GSH and GSSG. **c** Relative expression of *glutathione reductase* (*GR*) in *D. lanata* WT and *Dl P5βR-RNAi* knockdown shoots either untreated or BSO treated. RNA expression levels were calculated using the qPCR method by applying the 2^−ΔΔCT^ method with actin as the reference gene. The *y* axis denotes the normalized relative transcript accumulation of the *GR* in the individual lines indicated in the figure legend. Mean ± SEM are shown (*n* = 3). **d** Quantification of digoxigenin and digitoxigenin in *Dl* WT and *Dl P5βR-RNAi* knockdown lines treated with 0.3 mM BSO (Mean ± SEM are shown *n* = 3). Tukey’s test, *P > 0.05

The knockdown of either *DlP5βR*1 or *DlP5βR*2 in *D. lanata* led to a decrease of the 5β-cardenolide content in shoot cultures (Fig. 3d). This can be taken as evidence that PRISEs are indeed involved in cardenolide formation. Berglund and Ohlsson (1993) reported that glutathione (GSH) impairs cardenolide biosynthesis. Therefore, elevated glutathione levels could also be responsible for the reduced cardenolide levels observed in the *Dl P5βR-RNAi* lines. Berglund and Ohlsson (1993) further reported that buthionine sulfoximine (BSO), described as an inhibitor of glutamylcysteine synthetase and stimulated cardenolide biosynthesis in *D. lanata* tissue cultures. They also demonstrated that the stimulating effect of BSO on digitoxin accumulation was diminished by the simultaneous addition of GSH. In our experiment with *Dl* WT shoots, we detected no stimulating effect of BSO on cardenolide level. This can be explained by the fact that the shoot cultures used here already contained considerable amounts of cardenolides which was not the case in the tissues used by Berglund and Ohlsson (1993). We did, however, determine the compensating effect after treating the shoots with GSH (Fig. 4a). As GSH levels can influence cardenolide level, we continued in our experiments to estimate GSH pools in WT and *Dl P5βR-RNAi* lines. In addition, we measured the expression of glutathione reductase (*GR*), and took it as a surrogate for imbalances in glutathione metabolism. *GR* reduces glutathione disulfide to glutathione and contributes to maintain the reducing environment in the cell in this way. Compared to WT shoots all RNAi-mediated *P5βR* knockdown lines showed increased levels of GSH and a higher gene expression of *GR* (Fig. 4b, c). Treatment with BSO amplified *GR* expression in WT shoots 5 times compared to non-treated controls but not in *RNAi*-mediated *P5βR* knockdown lines (Fig. 4c).

After BSO treatment cardenolide formation could be restored in *Dl P5βR*2*-RNAi* lines but not in *DlP5βR*1-*RNAi* lines indicating that *P5βR*1 is more important for constitutive cardenolide biosynthesis than *P5βR*2 (Fig. 4d). However, *P5βR*2 expression can be increased considerably by various types of stress. In the *Dl P5βR1-RNAi* lines, cardenolide biosynthesis could not be restored by adding BSO, indicating that the reduced cardenolide level was not only caused by altered GSH levels, but correlated directly with reduced *P5βR1* expression (Fig. 4d). To the best of our knowledge, this is the first proof that *P5βR1* is indeed directly involved in β-cardenolide formation. For example, *VEP1*, a P5βR1 homologue from *Arabidopsis thaliana* expressed *in D. purpurea*, did not enhance 5β-cardenolide accumulation (Kairuz et al. 2020).

### Effects of 5β-pregnane-3,20-dione, progesterone and methyl vinyl ketone

Knowing that the successful knockdown of *P5βR in D. lanata shoots* caused a decrease in cardenolide level, we tried to restore cardenolide content by feeding 5β-pregnane-3,20-dione to WT and *Dl P5βR1-RNAi* lines. Interestingly, we observed a recovery of cardenolide content in *Dl P5βR1-RNAi* lines along with a reduction of glutathione levels. Cardenolide content in *Dl P5βR1-RNAi* lines reached around 2/3 (0.8 µmol × g^−1^ DW) of the cardenolide level of WT shoots. Still high GSH levels in *Dl P5βR2-RNAi* lines seem to continue disturbing cardenolide formation. Cardenolide levels increased here from around 0.3 µmol × g^−1^ DW to 0.5 µmol × g^−1^ DW. In WT shoots, feeding 5β-pregnane-3,20-dione did not influence cardenolide level (Suppl. Fig. S5 a, b).

Haussmann et al. (1997) administered various pregnanes to photomixotrophic, cardenolide-producing shoot cultures of *Digitalis lanata* as well as to cardenolide-free tissue cultures of the same plant. Depending on the pregnane precursor added the cardenolide content increased, but only in the mixotrophic shoot cultures. Studying effects of different pregnanes not only on WT shoots but also on RNAi knockdown lines similar to the study conducted by Haussmann et al. (1997) can give more detailed information on cardenolide formation in *Digitalis lanata* and will be part of future studies.

Since progesterone 5β-reductase activity was reduced in *Dl P5βR-RNAi* lines, progesterone may accumulate. Progesterone, a mammalian sex hormone, has been considered as a plant hormone (Iino et al. 2007). Assuming no feedback inhibition of progesterone biosynthesis or alternative metabolization, gene knockdown of progesterone-converting enzymes should lead to enhanced progesterone levels. We investigated the possibility of physiologically active progesterone being present by estimating progesterone levels using GC–MS and UPLC. We found that all *RNAi* lines contained less progesterone than the WT shoots and that cardenolide levels could not be restored by feeding exogenous progesterone to *Dl P5βR-RNAi* shoots (Suppl. Fig. S5c). Progesterone can also enhance the activity of antioxidant enzymes and increases GSH levels (Genisel et al. 2013). In our experiments, *glutathione reductase* (*GR*) expression was increased by a factor of 3 in *Dl* WT shoots and even by a factor 8 in all *Dl P5βR-RNAi* lines after 7 days of treatment with 0.3 mM progesterone (Suppl. Figure 5d), indicating again an imbalance of the redox state (endogenous GSH pool). This resulted in a disturbance of the progesterone metabolism and that might constitute a feedback regulation of progesterone biosynthesis. Feedback loops in pathways of specialized metabolism are known. For example, *AOP2*, a protein involved in aliphatic glucosinolate biosynthesis, mediated a feedback regulation in *A. thaliana* (Burow et al. 2015).

**Fig. 5:**
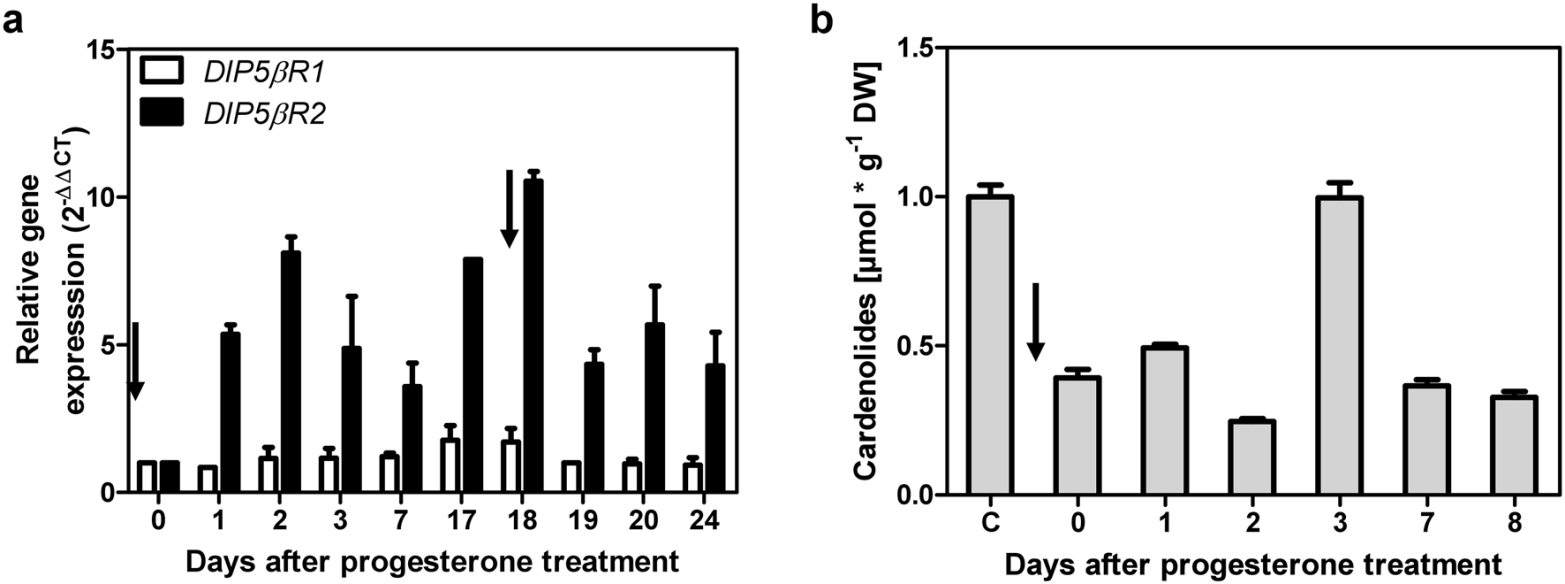
Expression of progesterone 5β-reductases and cardenolide in shoot cultures of *D. lanata* after progesterone treatment. **a** Relative RNA expression levels were calculated by qPCR method by applying the 2^−ΔΔCT^ method using *actin* as reference gene. The y axis denotes the normalized relative transcript accumulation of *DlP5βR1* and *DlP5βR2* genes after progesterone treatment indicated by black arrows. (Mean ± SEM are shown; n = 3). **b** Quantification of digoxigenin and digitoxigenin in *D. lanata* WT shoots after progesterone treatment indicated by black arrow. (Mean ± SEM are shown; *n* = 3)

When progesterone (0.16 mM) was fed to WT shoots, it was consumed completely after 7 days of cultivation. When progesterone was fed repeatedly in identical batches, it was consumed faster with every new batch indicating an increase in progesterone-converting processes within the cell (data not shown). This can be explained by the sixfold increased expression of *DlP5βR2* after the addition of progesterone, reaching a similar expression level as *DlP5βR1* but having a much higher activity for converting progesterone (Table 1)*. DlP5βR2* expression was boosted after adding a new batch of progesterone (Fig. 5a). This explained the accelerated conversion of exogenous progesterone in the fed-batch experiment (k_cat_ value for progesterone, Table 1). The expression of *DlP5βR1* was only slightly affected by the progesterone treatment (Fig. 5a). Cardenolide formation peaked 3 days after the addition of progesterone (Fig. 5b). This increase was paralleled by a transient increase of *DlP5βR*2 expression. Similar effects were reported by Pérez-Bermúdez et al. (2010) when investigating stress provoked by wounding, hydroperoxide and 1-aminocyclopropane-1-carboxylic acid (ACC), a precursor of the phytohormone ethylene in plants. Progesterone was reported to occur in many plant species (Janeczko and Skoczowski 2005; Pauli et al. 2010) and exogenous progesterone influenced plant growth (Bhattacharya and Gupta 1981). It has a positive impact on abiotic stress resistance in a broad range of crops (Janeczko et al. 2013; Genisel et al. 2013; Hao et al. 2019). We, therefore, considered the cardenolide formation observed in a developmental context as “constitutive” biosynthesis (connected to the constitutive expression of *DlP5βR*1) and “stress-induced” biosynthesis (connected to the inducible expression of *DlP5βR*2 (Fig. 5a)).

Exogenous [^14^C]-progesterone was demonstrated to be incorporated into cardenolides. Low incorporation rates of less than 1% were observed with most of the radioactivity detected in a complex, unidentified mixture of products (Eisenbeiß et al. 1999). It is possible that the excess of progesterone not used for cardenolide biosynthesis influenced other metabolic processes.

Being an active α,β-unsaturated carbonyl, progesterone is a reactive electrophile substance (RES). In order to separate stress effects from precursor effects, we tested the effect of methyl vinyl ketone (MVK) on *DlP5βR* gene expression.

**Fig. 6:**
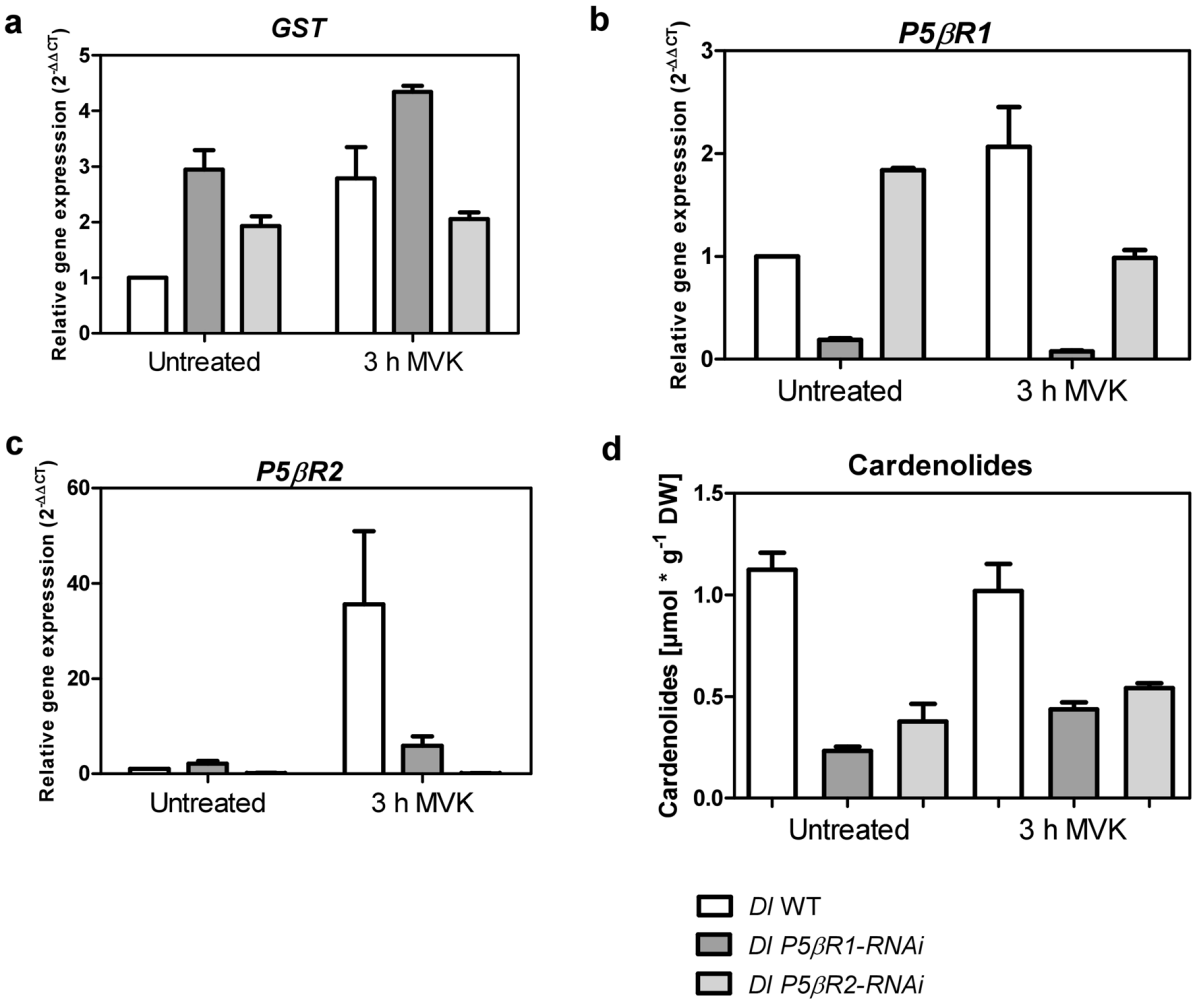
Effects of MVK on gene expression, stress response and cardenolide content in *Digitalis lanata* WT and *Dl P5βR-RNAi* knockdown shoots. **a**–**c** The relative RNA expression levels were calculated using the qPCR method by applying the 2^−ΔΔCT^ method with *actin* as the reference gene. The y axis denotes the normalized relative transcript accumulation of genes indicated at the top. X axis describes the MVK treatment conditions. Means ± SEM are shown (*n* = 3). Relative gene expression of *glutathione S-transferase* (*GST*) (**a**), *P5βR1* (**b**) and *P5βR2* (**c**), in *D. lanata* WT and *Dl P5βR-RNAi* knockdown shoots. **d** Quantification of digoxigenin and digitoxigenin in *D. lanata* WT and *Dl P5βR-RNAi* knockdown shoots treated with 2 µmol/L air volume MVK. Results are shown as mean ± SEM (*n* = 3)

Methyl vinyl ketone (MVK) is the simplest form of such an α,β-unsaturated carbonyl and is a peroxidation product of trienoic fatty acids, that is known to accumulate in plants in stress situations (Vollenweider et al. 2000; Alméras et al. 2003; Kai et al. 2012; Jardine et al. 2013). It is not influencing “constitutive” cardenolide biosynthesis (Munkert et al. 2014; Horn et al. 2020). Furthermore, MVK is a substrate for *P5βR*s and can be detoxified by them (Table 1; Suppl. Fig. S6 a; Munkert et al. 2015a, b). Investigating the physiological impact of PRISEs in general, *Dl* WT and *Dl P5βR-RNAi* lines were treated with MVK for 3 h (Munkert et al. 2014). *Glutathione S-transferase* (*GST*) was used as positive control for an MVK related effect, as it is a stress-related gene (Alméras et al. 2003) and is known to be able to detoxify MVK (Horiyama et al. 2014; Suppl. Figure 6 b). We here found that after MVK treatment *GST* gene was upregulated in *Dl* WT but remained almost unchanged in *Dl P5βR-RNAi* lines (Fig. 6a).

In WT shoots, the expression of *P5βR1* was only moderately enhanced (2.5 times) by MVK, whereas *P5βR2* expression increased 35-fold (Fig. 6b, c). It is assumed that MVK is detoxified rapidly to methyl ethyl ketone (MEK), a metabolite which does not influence *P5βR* gene expression (Suppl. Figure 7). In *Dl P5βR2-RNAi* lines, *P5βR1* expression was reduced to WT levels after MVK treatment, whereas *P5βR2* expression was only moderately enhanced by MVK in *Dl P5βR1-RNAi* lines. Most obvious was the dramatic increase of *P5βR2* expression in WT shoots but not in *DlP5βR1-RNAi*, which still contained the active P5βR2 enzyme. This indicates an altered stress response in *Dl P5βR1-RNAi* lines.

Similar to other studies (Munkert et al. 2014), cardenolide levels remained almost unchanged after MVK treatment in WT shoots (about 1 µmol g^−1^ DW) as well as in *Dl P5βR-RNAi* knockdown lines (0.3–0.5 µmol g^−1^) (Fig. 6d). Independently to cardenolide formation all in all, especially *P5βR2* expression is related to plant stress response and P5βR2 enzyme might favorable be involved in detoxification reaction.

## Conclusion

Our data led to the assumption that 5β-cardenolides are formed in a developmental process in which constitutively expressed *P5βRs* (*progesterone 5β-reductases*)*,* such as *P5βR1* are involved. Stress-induced *P5βR*s, such as *P5βR2* efficiently, yet transiently boosting cardenolide biosynthesis, may be beneficial in an immediate chemical defence scenario.

Despite possibly being involved in other physiological processes, the stress-inducible P5βR2 has an affinity to progesterone much higher than that of P5βR1 in this way channeling more progesterone into the cardenolide pathway. Using RNAi-mediated *P5βR1* and *P5βR2* knockdown shoot culture lines of *D. lanata*, we here provide solid evidence that P5βRs are not only involved in cardenolide biosynthesis but are also important for plants dealing with stress by detoxifying reactive electrophile species, such as MVK.

However, detailed studies of the expression of stress-related genes and cardenolide formation over longer periods of time after treating WT shoots and *Dl P5βR-RNAi* lines with, e.g., progesterone, 5β-pregnane-3,20-dione (its biosynthetic follow-up product) or additional RES are necessary to further assess the impact of *P5βR* knockdown on stress metabolism.

Supplemental material: supplemental material includes SDS-page and western blot analysis of heterologous expressed *Dl*P5βR2 in *E. coli*, verification and influence of stable transformation of and on *D. lanata* shoots as well as quantification and influence of progesterone and 5β-pregnane-3,20-dione in WT and RNAi lines. Tables listed primer pairs for qPCR analysis as well as verification of T-DNA integration in *D. lanata* genome. All sequences including accession numbers are also provided as Supporting Information.

## Supplementary Information

Below is the link to the electronic supplementary material.

Supplementary file1 (PDF 1290 KB)

## Abbreviations

BSO: Buthionine sulfoximine
GSH: Glutathione
GR: Glutathione reductase
GST: Glutathione S-transferase
ISY: Iridoid synthases
MVK: Methyl vinyl ketone
PRISE: Progesterone 5β-reductase/iridoid synthase-like enzyme
P5βR: Progesterone 5β-reductases
PO: Progesterone
PR: 5β-Pregnane-3,20-dione
RES: Reactive electrophile species
St5βR: Steroid 5β-reductase
