# Supplementary material for: RNAi-mediated gene knockdown of progesterone 5β-reductases in Digitalis lanata reduces 5β-cardenolide content
Source: Plant Cell Rep. 2021 Jun 19;40(9):1631–46. doi: 10.1007/s00299-021-02707-3 (PMC8376734; doi:10.1007/s00299-021-02707-3)

Supplemental Material:

RNAi-mediated gene knockdown of progesterone 5β-reductases in *Digitalis lanata* reduces 5β-cardenolide content and modifies stress responses.

Jan Klein[a], Elisa Horn[a], Mona Ernst[a], Tim Leykauf[a], Tamara Leupold[a], Maja Dorfner[a], Laura Wolf[a], Anastasiia Ignatova[a], Wolfgang Kreis[a], Jennifer Munkert[a,b]

[a]Department of Biology, University of Erlangen-Nuremberg, 91058 Erlangen, Germany.
[b]Corresponding Author: jennifer.munkert@fau.de

Figure legends:

Fig S1: **Identification of T-DNA insertion into the plant genome.** Results of PCRs with genomic DNA from wildtype (wt/ WT) and transgenic (1, 2) *Digitalis lanata* shoots. (–) negative water control, (+) positive control – vector control or respectively agrobacterium culture (*virD2*). (a) Results of PCR against *virD2*; (b) Results of PCR against *spec*. No bacterial DNA including plasmid signals were detected in wildtype nor in transgenic shoots. (c) Results of PCR against *nptII*; (d) Results of PCR against *GUS* gene. In both cases signals were detected in transgenic, but not in wildtype shoots. (e, f) *nptII*-expression in transgenic *D. lanata P5βR-RNAi* knockdown shoots confirmed by PCR against cDNA. Expression of selection gene *nptII* was verified by PCR with cDNA of wildtype and transgenic shoots and primer system *nptII.* No expression of *nptII* was detected in wildtype (WT).

Fig S2: **RNAi cloning strategy.** Schematic diagram of recombination reactions with pHellsgate8 vector. PCR products flanked by attB sites are recombined with pDONR221 vector in a BP clonase reaction. The resulting clone in pDONR221 can be recombined with pHellsgate8 in an LR clonase reaction.

Figure S3: **Heterologous expression of CDS of *Dl*P5βR2 in pDEST17 vector in *E. coli*.** (a) SDS-Page analysis of recombinant *Dl*P5βR2 (1). Purified r*Dl*P5βR2 protein has a size of about 43 kDa and was visualized with Coomassie-Brilliant-Blue R 250; (b) Immunoblot analysis of r*Dl*P5βR2 (2) using anti-His antibodies (primary) and anti-mouse IgG-peroxidase antibodies (secondary). Chemiluminescence was used for detection. (M) Marker.

Figure S4: Relative transcripts of *P5βRs* (*DlP5βR1* and *DlP5βR2*) in *D. lanata* WT shoots and shoots transformed with 679p935s-GusIo-rbs (VC). 679p935s-GusIo-rbs transformed shoots were used as control to exclude artificial effects created by agrobacteria transformation process. The relative RNA expression levels were calculated by qPCR method by applying the $2^{-\Delta\Delta CT}$ method using actin as reference gene. The y axis denotes the normalized relative transcript accumulation of the PRISE genes indicated in the x axis. (mean ± SEM; n = 3).

Figure S5: Progesterone and cardenolide levels in *Dl* WT and stable transformed shoots of *D. lanata* including progesterone and 5β-pregnane-3,20-dione treatment and estimation of GSH levels. (a,b) Effects of 5β-pregnane-3,20-dione (PR) treatment on cardenolide level in *D. lanata* WT shoots (a) and on GSH pool in *Dl* WT and *Dl P5βR-RNAi* knockdown lines calculated by measuring t-GSH and GSSG (b). (c) Progesterone (PO) was identified by GC-MS and quantified by UPLC.(d) Quantification of digoxigenin and digitoxigenin in *D. lanata* WT and *P5βR-RNAi* knockdown shoots after progesterone treatment. (e) Relative expression of *glutathione reductase* (*GR*) in *D. lanata* WT and *Dl P5βR-RNAi* knockdown shoots either untreated or progesterone treated. RNA expression levels were calculated using the qPCR method by applying the $2^{-\Delta\Delta CT}$ method with actin as the reference gene. The y axis denotes the normalized relative transcript accumulation of the *GR* in the individual lines indicated in the figure legend. (f) Decrease of progesterone and 5β-pregnane-3,20-dion level after feeding in the medium. (Mean ± SEM are shown; n = 3).

Figure S6: Detoxification mechanism of reactive electrophile species (RES) *in planta*. Detoxification e.g. of MVK can either happen by reduction ((a); Chapellin et al., 2019) or via GST-catalyzed reaction forming a glutathione conjugate ((b); Yin et al., 2017)

Figure S7: Gene expression of *Act*, *P5βR1* and *P5βR2* in *D. lanata* WT shoots. PCR results for gene expression of *Act*, *P5βR1* and *P5βR2 in D. lanata* WT shoots after treatment with 2 µmol/ L airvolume MVK or MEK diluted in potable water for 3 h. Control shoots were treated with pure potable water.

Supplement Tables:

Table S1: Primers used for the verification of T-DNA integration into *Digitalis lanata* genome.

Name	Sequence in 5´-3´direction	T_A in ° C
nptII for:	TGA ATG AAC TGC AGG ACG AG	65
nptII rev:	AAT ATC ACG GGT AGC CAA CG	
GUS for:	GCA AAG TGT GGG TCA ATA AT	55
GUS rev:	ATC ACA CTC TGT CTG GCT TT	
SmR for:	GGT CCA GAA CCT TGA CCG AA	57
SmR rev:	CCA CGG AAT GAT GTC GTC GT	
virD2 for:	ATG CCC GAT CGA GCT CAA GT	55
virD2 rev:	CCT GAC CCA AAC ATC TCG GCT GCC CA	

Table S2: Primer used for quantification of gene expression by qPCR in *Digitalis lanata*. Analyzed genes: *actin*, *P5βR1*, *P5βR2*, *glutathione-reductase* (*GR*), and *glutathione S-transferase* (*GST*)

Name	Sequence in 5´-3´direction	T_A in ° C
EH_qDlAct for:	ATT CAG ATG CCC AGA AGT	62
EH_qDlAct rev:	GGA GAT CCA CAT CTG CTG GAA	
JK_qDlP5βR1 for:	TGC AAA CAC GAG GGA AAG GT	66
JK_qDlP5βR1 rev:	AAG CCA TGC TCC TTG CTC TT	
JK_qDlP5βR2 for:	CTG CAG GAC ACA AAA CGG TG	66
JK_qDlP5βR2 rev:	TCG TCC CAT ACC GAG TCC TT	
JK_qDlGR1 for:	GGT AGG GCT CCA AAC ACG AA	66
JK_qDlGR1 rev:	TCC TCG CTG AGA CCA ACA AC	
JK_qDlGST1 for:	GGT CCA TGG CAA CCC TAT CT	62
JK_qDlGST1 rev:	GCC TCA ACT TCA AGC CAC AC	

Sequences:

>*D. lanata* GST (MT948956)

ATGGCAATCAAGGTCCATGGCAACCCTATCTCTGCTCCAACAAGGAGAGTTCTTCTGTGTCTAGCAGAGAAAGAT
GTCGAATACGAATATGTTCACATAGATCTATTTACTGGTCAACACAAGAAAGAGCCCTTCATTTCAATCAACCCA
TTTGGTCAAGTTCCAGGTTTTGAAGATGGAGACTTGAATCTATTTGAATCAAGGGCAATCACCAAGTACATTGCT
CATGCATATGCTGACAAGGGAATTCCACTATTATCTGAAGATCCAAAGAAGATGGGAATCATCTCAGTGTGGCTT
GAAGTTGAGGCCCAAAGATTTGAAGCTGCAGGGCAGAAGCTTAACATTGAAATAGTGATACATCCACTGATTGGG
CAGACCACTGATGAGGCCAAAGTGGAACAACTCCAGGCCCAACTGGCCTCAGTTCTTGATGTGTACGAAGCCCGG
TTGGCCCAATCCAAGTACTTGGCAGGAGACTCCTACACACTTGCTGATCTTCATCATGTCCCTGTCATCAACAAC
TTGATGCAGACAAAGATCAAGACATTGTTCGATGAGCGTCCCCACGTGAGTGCGTGGTGCACTGATCTCTTGGCG
CGTCCTGCTTGGCAGAAGGTTCTTGCATGGCTCAATAGTCAGTAA

>*D. lanata* Act-Fragment (MT948955)

TCCAATCCAAACACTGTACTTCCTCTCTGGTGGTGCAACAACCTTTATCTTCATGCTGCTAGGAGCCAATGCTGT
GATTTCCTTGCTCATACGGTCAGCAATACCAGGGAACATGGTTGAACCACCACTGAGGACAATATTACCATAGAG
ATCCTTCCTAATATCAACATCACACTTCATGATGGAGTTGTACGTAGTCTCATGGATTCCAGCTGCTTCCATGCC
AGTTAAAGAAGGCTGGTATAGGACTTCTGGGCATCTGAATATCTTTCTAGAAGATCTCCTACAATATTCTCAGCT
GCCATGGAAAATCGATGTTCTTCTTTTATTCTCTCAAGATTTTCAGGCTGTATATTAAAACTTATATTAAGAACT
ATGCTAACCACCTCATCAGGAACCGTTGTAGGTGGCGTGGGTTTTCTTGGCAATCGACTCTCATGAAAACTACGA
GCTAAATATTCAATATGTTCCTCTTGACCAACTTTATTCTGCATTTTTTTTGAACGAGGTTTAGAGCAAGCTTCA
GGAAAACTGAGACAGGAATTTTATTAAAAATTTAAATTTTGAAGAAAGTTCAGGGTTAATAGCATCCATTTTTTG
CTTTGCAAGTTCCTA

>*D. lanata* P5βR1-Fragment(AY585867.1)

TGCAAACACGAGGGAAAGGTTTTGAGGTTTACTGGTTGTAAGGCTGCGTGGGATGGGTACTCGGATTGCTCTGAT
GCGGATTTGATAGCGGAGCATCATATTTGGGCTGCAGTGGATCCTTATGCAAAAAACGAGGCCTTTAATGTGAGT
AATGGAGATGTGTTTAAATGGAAGCATTTTTGGAAGGTGTTGGCGGAGCAGTTTGGAGTAGGGTGTGGAGAGTAT
GAAGAAGGGGTGGATTTGAAATTGCAGGATTTAATGAAGGGGAAGGAGCCGGTTTGGGAGGAAATCGTGAGGGAG
AATGGATTGACACCTACGAAACTGAAGGATGTCGGAATTTGGTGGTTTGGTGATGTTATACTTGGGAATGAGTGT
TTCCTGGATAGTATGAACAAGAGCAAGGAGCATGGCTT

>D. lanata P5βR2-Fragment(HM210089.1)

CTGCAGGACACAAAACGGTGCTCTAACATGGACAGTCCATCGCCCCGCACTAATTTTCGGGTTCTCACCATGTAG
TTTGATGAACATAGTCGCAACGCTAAGTGTTTATGCCGCGATTTGCAAATATGAGAACAAGCCGTTGGTGTATAC
CGGGACACAAACGTCGTGGAATTGTTTAGTGGATGCTGTGGATTCGGATTTGTTATCAGAGCACTTGGTATGGGG
TGCGATCAGCCCAAATGCGAAGAACCAAGCTTTCAATATCAACAATGGCGACGTTTTTAAATGGAAACATATATG
GAAAGTGTTGGCGGAGCAGCTTCAGCTTGAGATTGAGTTTGTGGGTTATGAAGGTAAGGAGCCGGTGTCTTTGGA
GGGTCTCATGAAGGATAAGGACTCGGTATGGGACGA

>*D. lanata* GR-Fragment(MT948958)

GGTAGGGCTCCAAACACGAAAAGGTTAAATCTGGAATCTGTAGGGGTTGAACTCGATAAATATGGAGCTGTGGTG
GTTGATGATTACTCTCGAACCAAAGTACCTAGCATATGGGCCATAGGTGATGTTACAAACCGTATGAATCTTACT
CCTGTTGCCTTAATGGAAGGAACCTGTTTTGCCAAAACTGTGTTCGGTGGGCAGCCTTCCAAACCAGACTACGAC
CATATTCCTTGCGCTGTCTTCTGCATCCCACCACTTTCAGTTGTTGGTCTCAGCGAGGA

>*D. lanata* GST-Fragment

GGTCCATGGCAACCCTATCTCTGCTCCAACAAGGAGAGTTCTTCTGTGTCTAGCAGAGAAAGATGTCGAATACGA
ATATGTTCACATAGATCTATTTACTGGTCAACACAAGAAAGAGCCCTTCATTTCAATCAACCCATTTGGTCAAGT
TCCAGGTTTTGAAGATGGAGACTTGAATCTATTTGAATCAAGGGCAATCACCAAGTACATTGCTCATGCATATGC
TGACAAGGGAATTCCACTATTATCTGAAGATCCAAAGAAGATGGGAATCATCTCAGTGTGGCTTGAAGTTGAGGC

Fig. S1

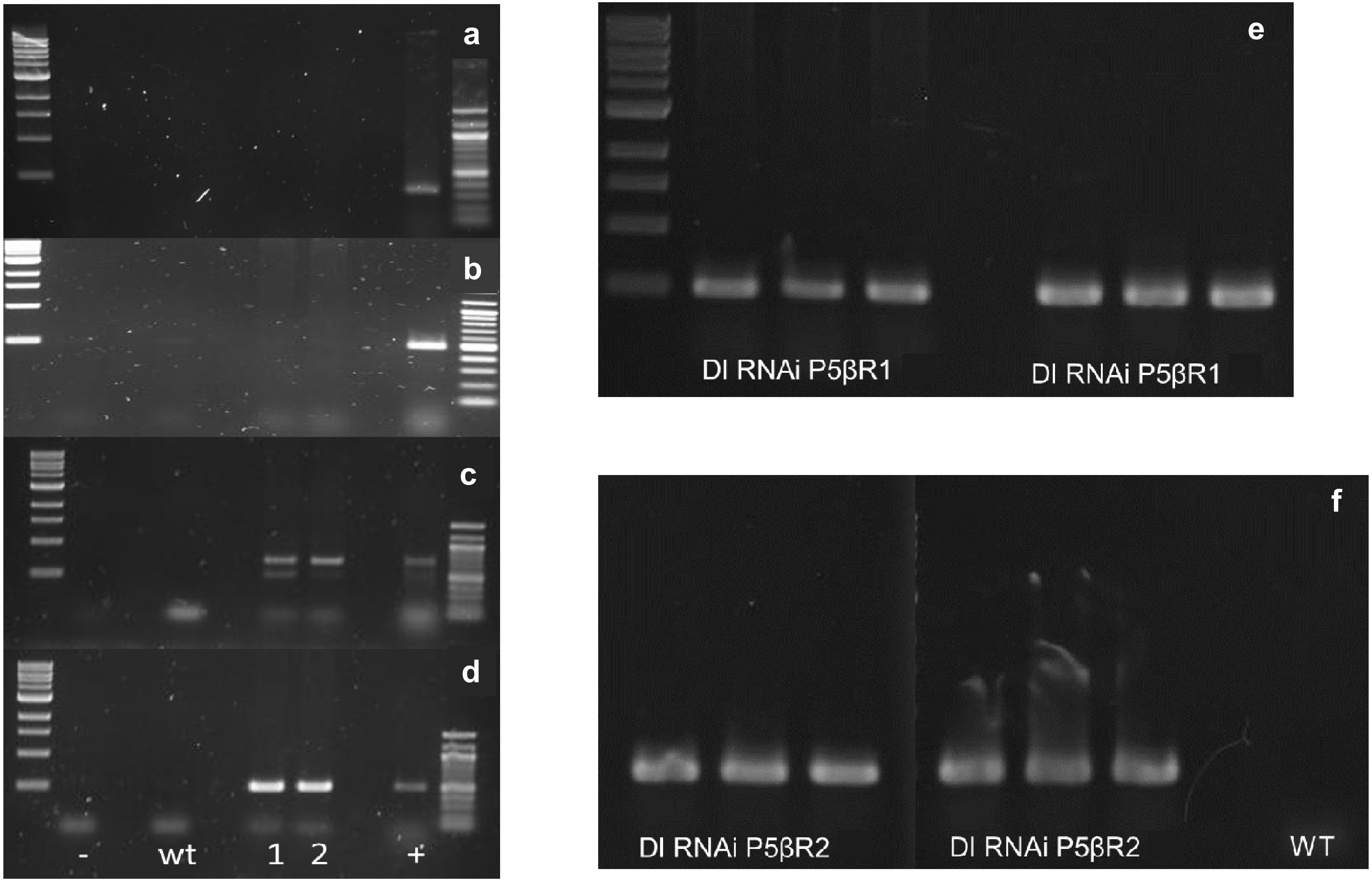
a
b
c
d
-
wt
1
2
+
e
DI RNAi P5βR1
DI RNAi P5βR1
f
DI RNAi P5βR2
DI RNAi P5βR2
WT

Fig. S2

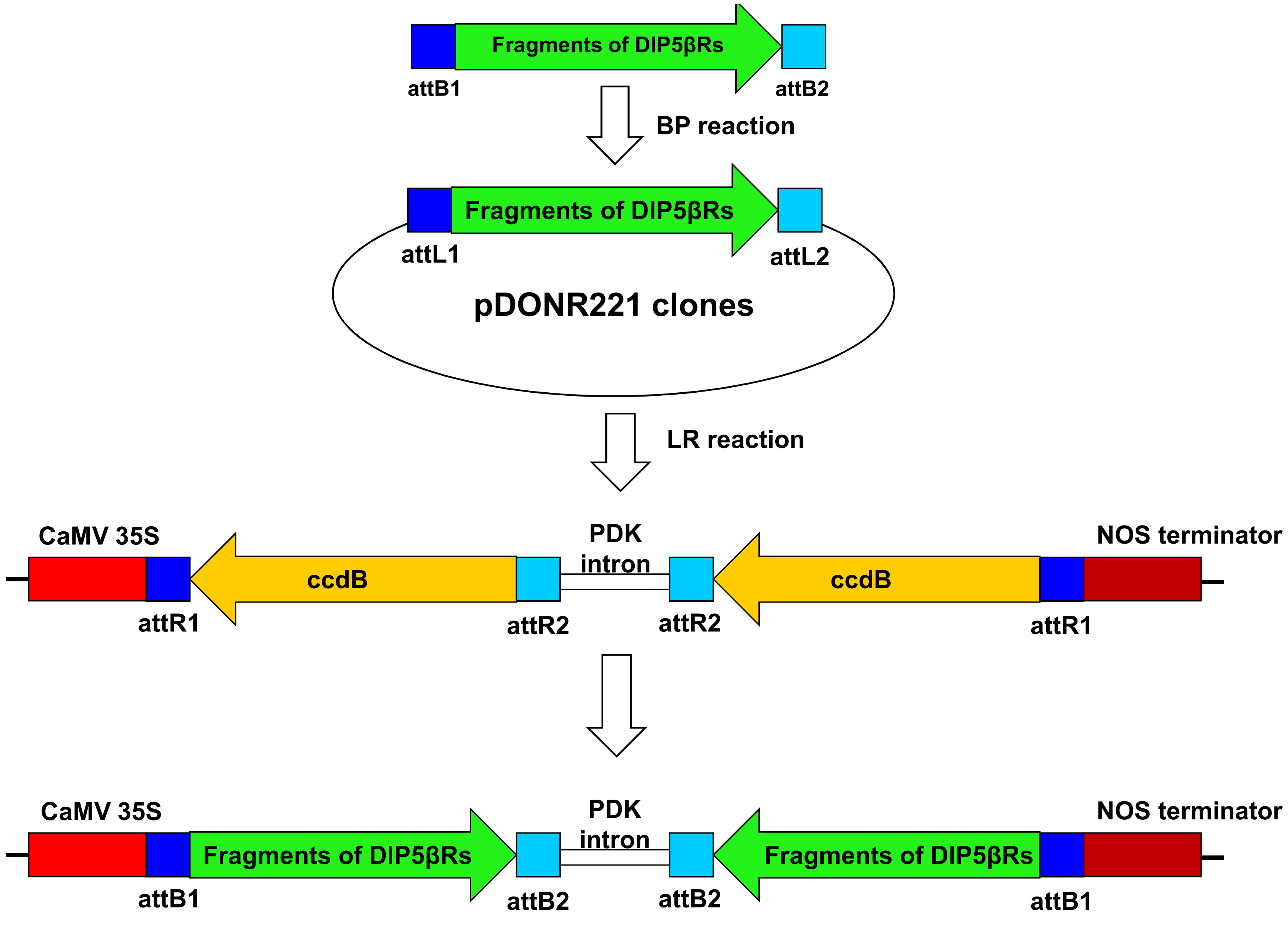

Fragments of DIP5βRs
attB1
attB2
BP reaction
Fragments of DIP5βRs
attL1
attL2
pDONR221 clones
LR reaction
CaMV 35S
ccdB
PDK
intron
ccdB
NOS terminator
attR1
attR2
attR2
attR1
CaMV 35S
Fragments of DIP5βRs
PDK
intron
Fragments of DIP5βRs
NOS terminator
attB1
attB2
attB2
attB1

Fig. S3

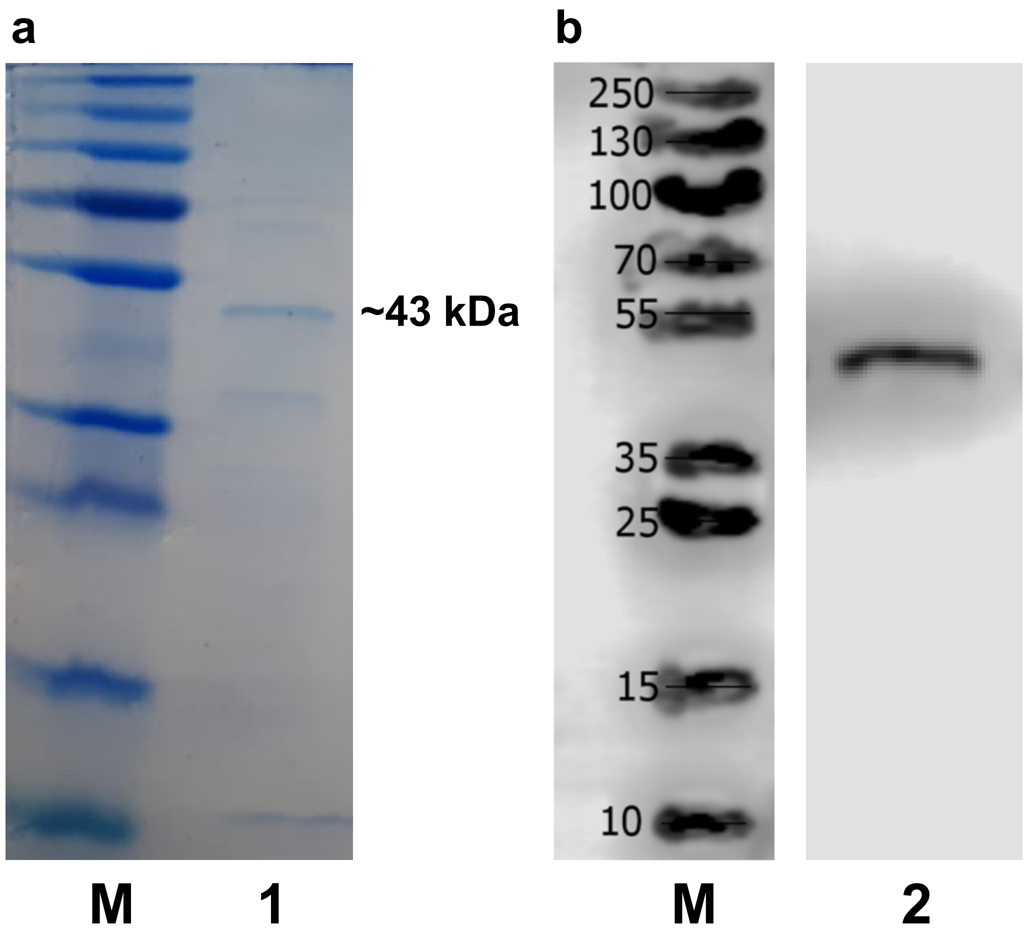

Fig. S4

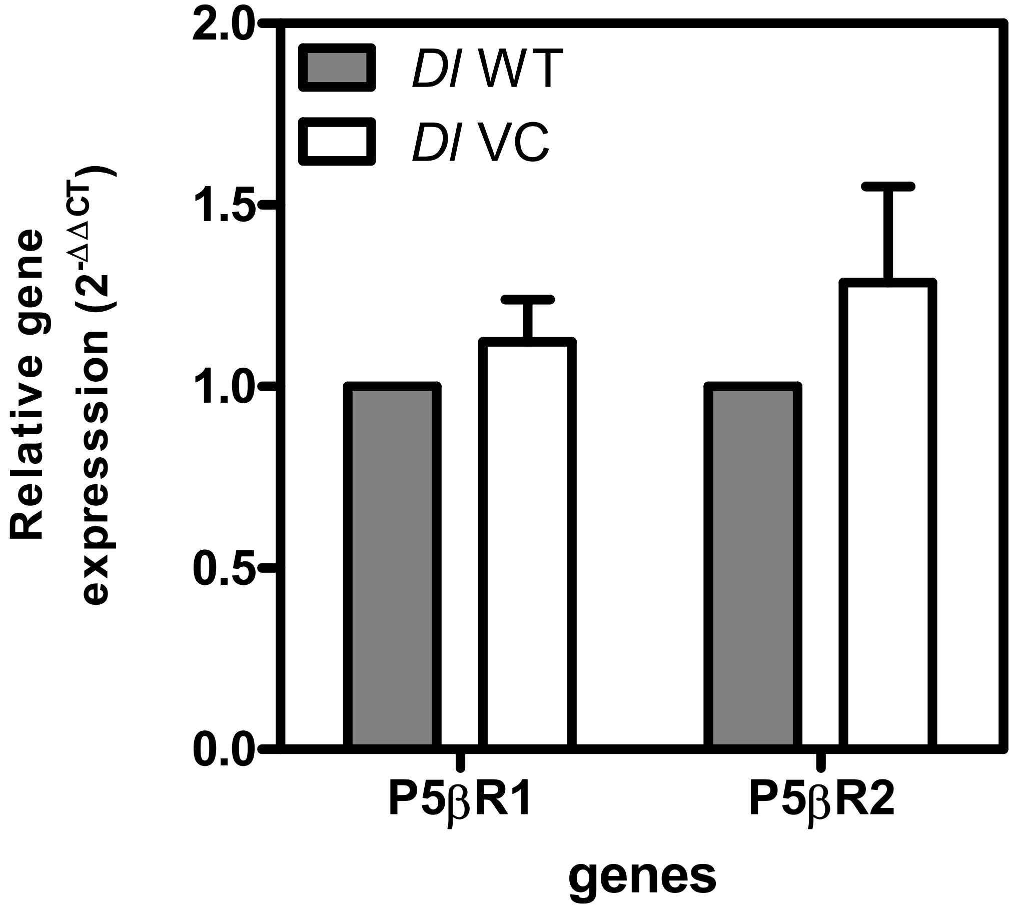

Dl WT
Dl VC
Relative gene expression (2-ΔΔCT)
2.0
1.5
1.0
0.5
0.0
P5βR1
P5βR2
genes

Fig. S5

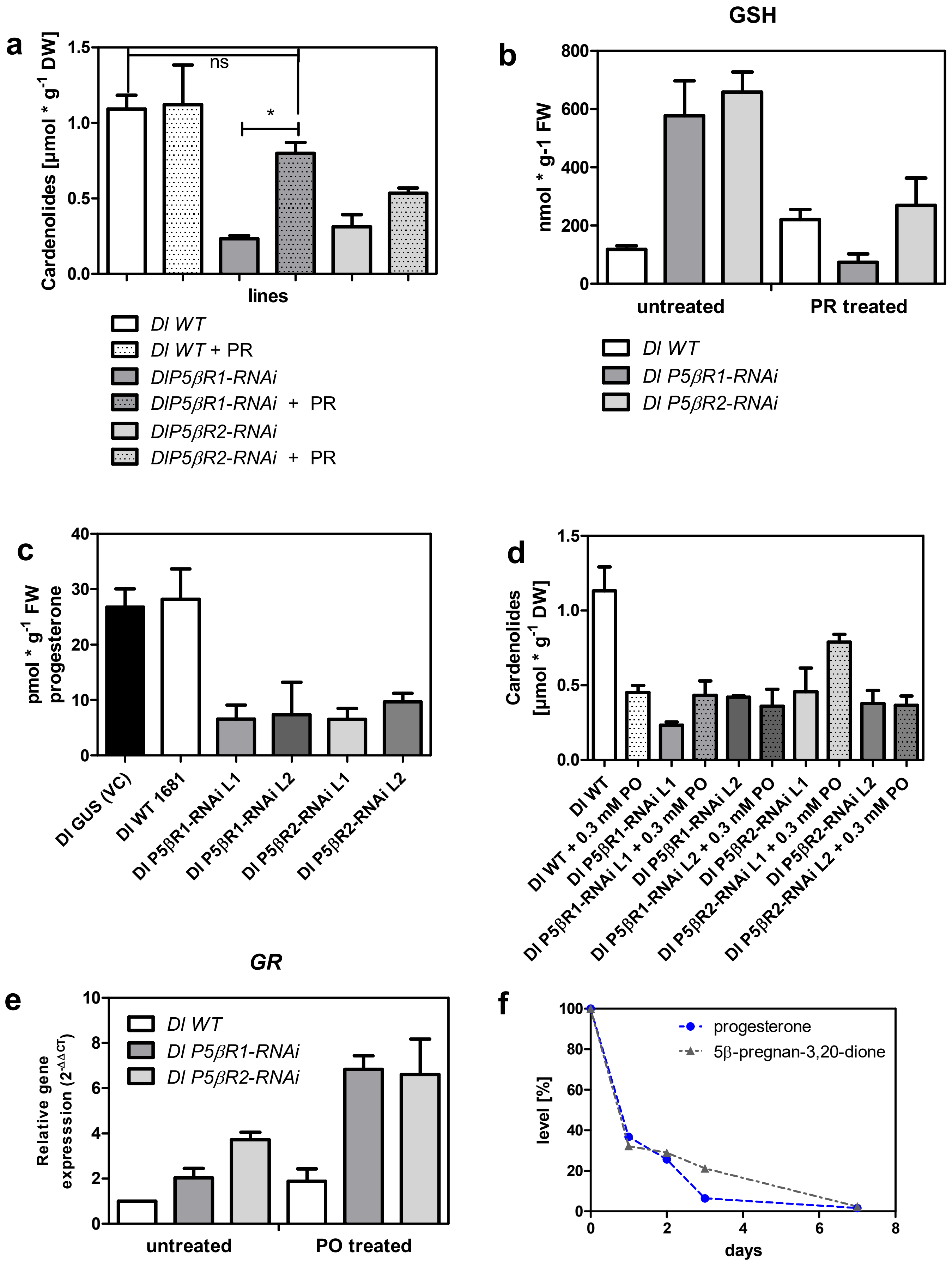

a
Cardenolides [µmol * g-1 DW]
0.0
0.5
1.0
1.5
ns
*
lines
Dl WT
Dl WT + PR
DlP5βR1-RNAi
DlP5βR1-RNAi + PR
DlP5βR2-RNAi
DlP5βR2-RNAi + PR
b
GSH
nmol * g-1 FW
0
200
400
600
800
untreated
PR treated
Dl WT
Dl P5βR1-RNAi
Dl P5βR2-RNAi
c
pmol * g-1 FW progesterone
0
10
20
30
40
Dl GUS (VC)
Dl WT 1681
Dl P5βR1-RNAi L1
Dl P5βR1-RNAi L2
Dl P5βR2-RNAi L1
Dl P5βR2-RNAi L2
d
Cardenolides [µmol * g-1 DW]
0.0
0.5
1.0
1.5
Dl WT
Dl WT + 0.3 mM PO
Dl P5βR1-RNAi L1
Dl P5βR1-RNAi L1 + 0.3 mM PO
Dl P5βR1-RNAi L2
Dl P5βR1-RNAi L2 + 0.3 mM PO
Dl P5βR2-RNAi L1
Dl P5βR2-RNAi L1 + 0.3 mM PO
Dl P5βR2-RNAi L2
Dl P5βR2-RNAi L2 + 0.3 mM PO
e
GR
Relative gene expresssion (2-ΔΔCT)
0
2
4
6
8
10
Dl WT
Dl P5βR1-RNAi
Dl P5βR2-RNAi
untreated
PO treated
f
level [%]
0
20
40
60
80
100
progesterone
5β-pregnan-3,20-dione
0
2
4
6
8
days

Fig. S6

a

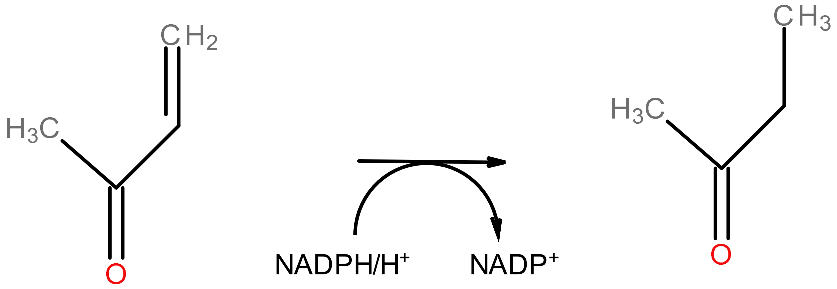
CH2
H3C
O
NADPH/H+
NADP+
CH3
H3C
O

b

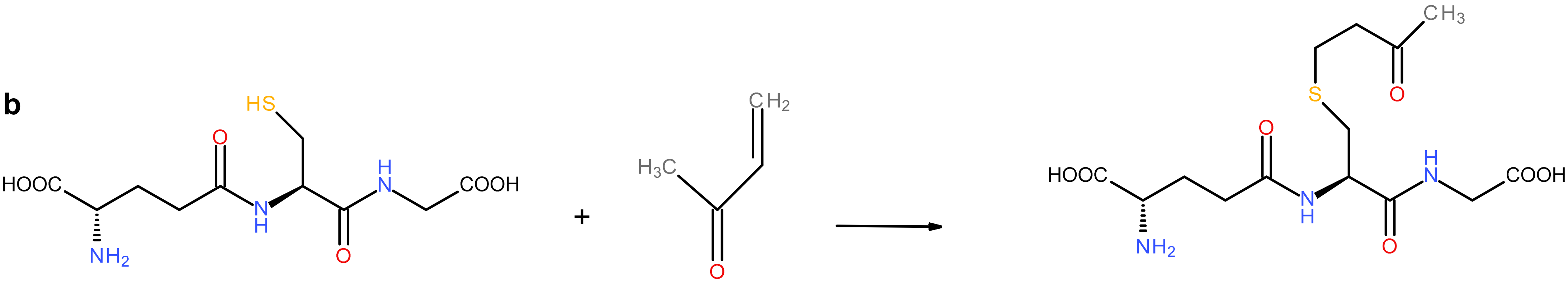
HS
O
HOOC
N
H
H
N
COOH
NH2
O
+
CH2
H3C
O
CH3
S
O
O
HOOC
N
H
H
N
COOH
NH2
O

Fig. S7

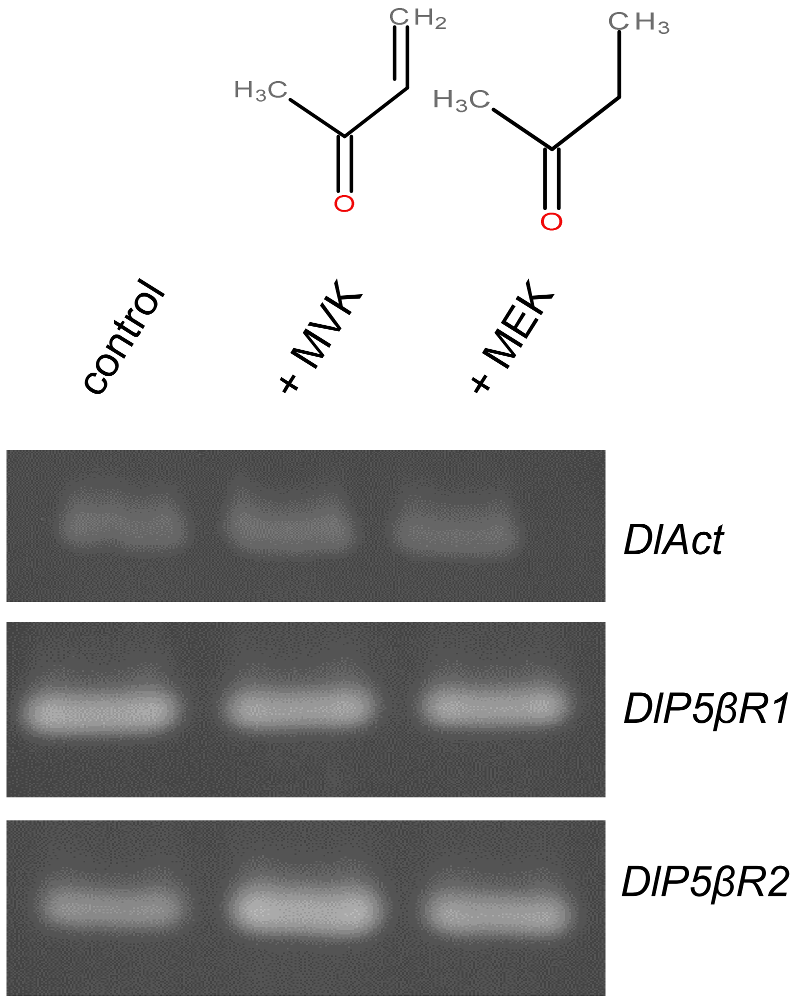

Supplement: Supplementary file 1 — Supplementary file1 (PDF 1290 KB) [file 299_2021_2707_MOESM1_ESM.pdf]
